# From Covalent Traps to Fluorescent Beacons: The Expanding Arsenal of Chemical Probes for Studying Ubiquitin and Ubiquitin‐Like Proteins

**DOI:** 10.1002/anie.202520118

**Published:** 2026-02-11

**Authors:** Saibal Chanda, Wenshe Ray Liu

**Affiliations:** ^1^ Department of Biochemistry and Biophysics College of Agriculture and Life Sciences Texas A&M University College Station Texas 77843 USA; ^2^ Texas A&M Drug Discovery Center and Department of Chemistry College of Arts and Sciences Texas A&M University College Station Texas 77843 USA; ^3^ Institute of Biosciences and Technology and Department of Translational Medical Sciences School of Medicine Texas A&M University Houston Texas 77030 USA; ^4^ Department of Cell Biology and Genetics School of Medicine Texas A&M University College Station Texas 77843 USA; ^5^ Department of Pharmaceutical Sciences Irma Lerma Rangel College of Pharmacy Texas A&M University College Station Texas 77843 USA

**Keywords:** activity‐based probe, activity‐based protein profiling, protein ligation, ubiquitin, ubiquitin‐like protein

## Abstract

Ubiquitin (Ub) and ubiquitin‐like proteins (Ubls) orchestrate diverse cellular processes through reversible post‐translational modification of target proteins. Their conjugation is governed by a cascade of E1 activating, E2 conjugating, and E3 ligating enzymes, while deconjugation is mediated by deubiquitinases (DUBs) and Ubl‐specific proteases. Profiling the catalytic activity of these enzymes is essential for understanding the dynamics and specificity of Ub/Ubl signaling. Activity‐based probes (ABPs) have emerged as powerful tools to covalently label active enzymes through electrophilic warheads that target catalytic residues. Unlike conventional affinity‐based approaches, ABPs capture functional states of enzymes in complex biological systems. This review provides a comprehensive analysis of ABPs designed for the Ub/Ubl signal pathways, encompassing probes for Ub, SUMO, NEDD8, ISG15, FAT10, UFM1, URM1, Atg8‐family modifiers, and FUBI (MNSFβ). We discuss key elements of probe design, including recognition domains, electrophilic warheads (e.g., vinyl sulfones, vinyl methyl esters, propargylamine, azapeptide esters), and detection tags. Particular emphasis is placed on emerging azapeptide ester‐based probes, which structurally mimic native enzyme‐substrate intermediates and offer high selectivity and reactivity. ABPs targeting E1, E2, and HECT/RBR E3 ligases are also highlighted, expanding their utility beyond classical DUB profiling. We further compare warhead chemistries, enzyme selectivity, and labeling strategies, and examine structural insights derived from probe‐enzyme complexes. Collectively, these tools have transformed our ability to interrogate Ub/Ubl‐regulating enzymes in vitro and in cells. The review concludes with perspectives on next‐generation probe development, including cell‐permeable designs, spatiotemporal control, and applications in systems biology and drug discovery.

## Graphical abstract

A large variety of chemistry‐based ubiquitin probes have been developed.

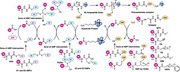

## 1 Introduction

**FIGURE 1:**
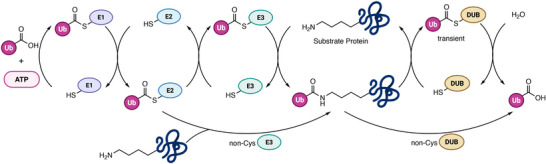
The ubiquitin conjugation/deconjugation cascade. The E1‐E2‐E3 cascade mediates the covalent attachment of ubiquitin (Ub) to a substrate protein, the process begins with an ATP dependent activation of Ub by an E1 activating enzyme, forming E1‐Ub thioester. The activated Ub is then transferred to E2 conjugating enzyme via transthiolation and finally ligated to the substrate via E3 ligase. Deubiquitinases (DUBs) catalyze the reverse process. Both E3 ligases and DUBs can be either cysteine‐dependent or cysteine‐independent. In cysteine‐based DUBs, ubiquitin forms only transient covalent intermediates that are rapidly hydrolyzed. Within this pathway, several steps serve as zones of activity‐based probes intervention, where tailored probes can covalently capture active enzymes for functional profiling.

Ubiquitin (Ub) and ubiquitin‐like proteins (Ubls) are small protein modifiers that regulate many essential cellular processes by reversibly attaching to target proteins [1]. These post‐translational modifications, known as ubiquitination and Ubl modifications, orchestrate protein degradation, localization, and complex formation, and their proper controls are critical [2, 3]. The principal function of ubiquitination is to label proteins for degradation via the proteasome, a mechanism fundamental to the regulation of diverse cellular pathways [4]. The dysregulation of the ubiquitin system contributes to diseases such as cancer and neurodegeneration [5]. Ubiquitin is typically conjugated to proteins via an isopeptide bond with a lysine side chain, though it can also attach to the *N* terminus through a peptide bond. Ubiquitination can halt after the addition of a single ubiquitin (monoubiquitination) or ubiquitin itself may be polyubiquitinated, generating chains through seven lysines (K6, K11, K27, K29, K33, K48, K63) or the N‐terminal methionine (M1) [6]. Among these, K48‐linked chains are the most studied, directing proteins to proteasomal degradation [7]. Other linkages generally mediate signaling—for example, M1‐linked (linear) chains are crucial in immune signaling pathways [8, 9]. Ubiquitination is mediated by an enzymatic cascade consisting of an E1 activating enzyme (Figure 1), an E2 conjugating enzyme, and an E3 ligase, which collaborate to attach Ub to a substrate protein lysine residue, often forming an isopeptide bond [10]. This modification is reversible and can be removed by deubiquitinases (DUBs), thereby maintaining the dynamic balance of signaling. Most Ubls are introduced to their target proteins via similar E1‐E2‐E3 cascades and removed by Ubl‐specific proteases. The human genome encodes ∼100 DUBs across seven different families (USP, UCH, OTU, MJD, MINDY, ZUP1 and JAMM) and dozens of Ubl‐specific proteases (e.g., SENPs for SUMO), underscoring the complexity of the Ub/Ubl signaling network [11, 12].

Characterizing activities of enzymes in E1‐E2‐E3 cascades is typically challenging in a complex cellular environment. Traditional assays (e.g., fluorogenic peptide substrates) measure activity but cannot easily attribute it to specific enzymes when many family members are co‐expressed. Activity‐based probes (ABPs) have emerged as powerful tools to profile these enzymes by covalently labeling active enzymes in an activity‐dependent manner [13, 14]. This approach is particularly valuable for identifying active enzymes within complex samples [15]. When combined with diverse covalent ubiquitin probes, they have enabled the characterization of functionally active cysteine enzymes, primarily DUBs, across the ubiquitin cascade in both tissues and disease‐derived cells [16, 17, 18]. An ABP is a mechanism‐inspired molecule that typically mimics an enzyme's natural substrate but carry a reactive electrophile (“warhead”) at the position of the originally cleaved bond to trap the enzyme's active‐site nucleophile, forming a stable covalent enzyme‐probe adduct. This allows direct detection of the active enzyme itself, rather than just its identity. Indeed, most DUBs and many conjugation enzymes utilize catalytic cysteine residues, making them ideal targets for cysteine‐reactive ABPs. Early studies demonstrated that Ub‐derived ABPs can irreversibly label active DUBs in cell lysates, enabling the discovery of new DUBs (e.g., identifying proteasome‐associated USP14) and the analysis of DUB regulation and inhibitor engagement [19]. The success of DUB probes has inspired extension of the approach to other steps in the cascade involving E1, E2, and E3 enzymes that are not proteases but often harbor catalytic cysteines and can be targeted by covalent probes.

In this review, we provide a comprehensive overview of activity‐based probes for Ub and Ubl pathways. We discuss probe design principles and mechanisms, review probes developed for DUBs and Ubl‐specific proteases, and survey ABPs on their targeting of the ubiquitin conjugation system. We compare the various probes’ specificities and applications and finally offer an outlook on future developments. Throughout, we highlight how these chemical tools have advanced our understanding of the ubiquitin and Ubl systems and enabled profiling of enzyme activities.

## 2 Design and Mechanisms of Ub/Ubl ABPs

### 2.1 General Architecture of ABPs

Activity‐based probes for Ub/Ubl enzymes share a common architectural blueprint comprising of three key elements:

#### 2.1.1 Recognition Element (Targeting Scaffold)

This provides specificity by engaging the target's substrate‐binding sites. In Ub/Ubl ABPs, the recognition element is often a full‐length ubiquitin or Ubl protein (or a peptide fragment thereof) that mimics a natural substrate of an enzyme. This ensures the probe binds in the enzyme's active site like a genuine substrate. In some cases, alternative targeting groups (small‐molecule inhibitors or peptides) have been used to target specific enzymes, but ubiquitin/Ubl‐based scaffolds are most common for broad profiling.

#### 2.1.2 Reactive Group (“Warhead”)

Typically, an electrophile is positioned at the site corresponding to the scissile amide bond between Ub/Ubl and its target protein. The warhead is designed to react with an active‐site nucleophile (usually a cysteine) in an enzyme's catalytic center, forming a covalent bond. This reaction occurs in an enzyme‐catalyzed fashion (often following initial binding and attack as in normal catalysis) so that only bona fide active enzymes are labeled. The choice of warhead is critical for probe reactivity and specificity, and various electrophiles have been employed.

#### 2.1.3 Reporter Tag (Detection Handle)

A reporter tag is a chemical tag that enables visualization or enrichment of the labeled enzyme. Common tags include fluorophores (for in‐gel fluorescence scanning), affinity handles such as biotin or FLAG/HA epitopes (for pull‐down and immunoblot detection), or small bioorthogonal groups (alkyne or azide) for two‐step labeling via click chemistry. In some large ABPs (e.g., full‐length Ub probes), the mass addition (∼8.5 kDa) itself causes a gel shift of the enzyme, allowing detection by SDS‐PAGE without an additional tag. Two‐step tagging (introducing a minimal alkyne/azide on the probe and adding a fluorescent/biotin tag post‐labeling) is often used when a bulky tag on the probe would reduce cell permeability or enzyme reactivity.

**FIGURE 2:**
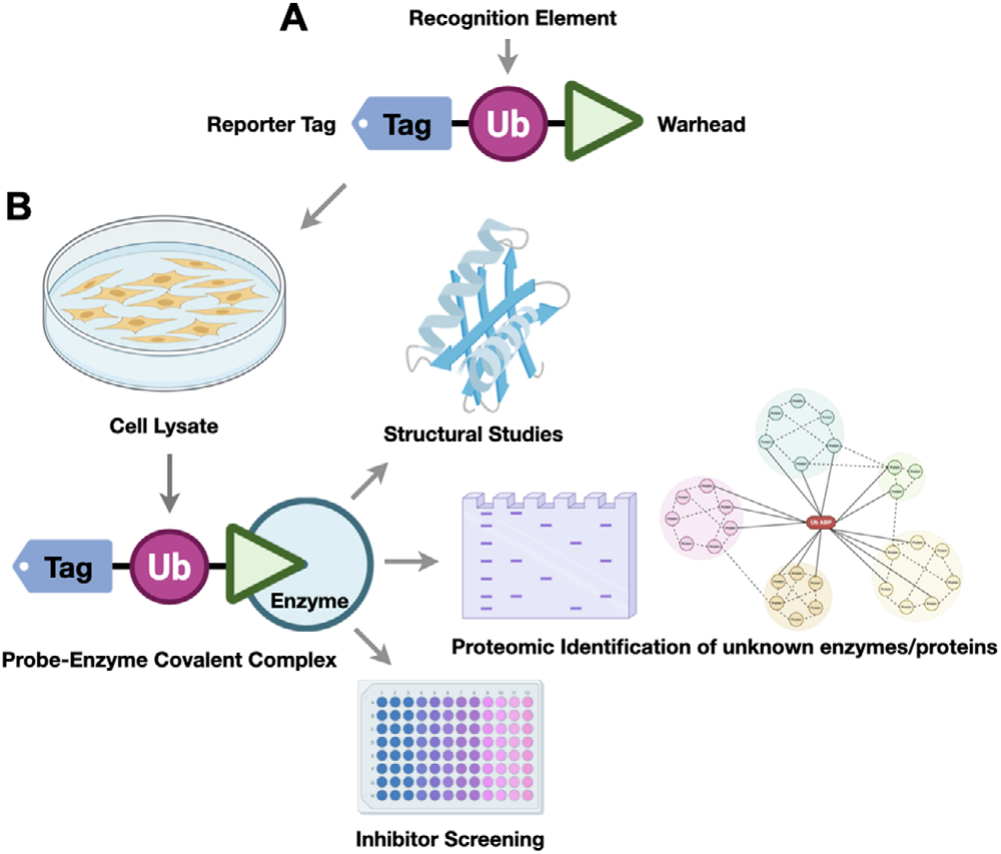
General design and applications of ubiquitin activity‐based probes. (A) Schematic representation of a Ub‐ABP, consisting of three components: a reporter tag (for detection or enrichment), an Ub recognition element and a warhead (reactive electrophile) that covalently traps target enzymes. (B) Downstream applications of Ub‐ABPs. Upon incubation with cell lysates, the probe forms a covalent complex with target enzymes, enabling: (i) structural studies of probe‐enzyme interactions, (ii) proteomic identification of novel enzymes or binding partners, and (iii) high‐throughput inhibitor screening. Collectively, these approaches allow both mechanistic insights and discovery of therapeutic modulators in the ubiquitin system.

Figure 2 presents a schematic illustration of a ubiquitin‐based activity‐based probe (Ub‐ABP), composed of three elements: a reporter tag for detection or enrichment, a ubiquitin recognition module (monoUb or diUb for specificity), and a reactive warhead that covalently captures target enzymes. An activity‐based probe thus “locks” the enzyme during catalysis, converting it into a covalent enzyme‐probe complex that can be detected. This strategy differs from conventional substrate assays in that the enzyme is irreversibly modified, enabling its isolation and identification. By virtue of covalent trapping, ABPs allow attribution of activity to specific enzymes even in a mixture, overcoming the challenge of overlapping specificities among DUBs or ligases. Broad‐spectrum ABPs, in combination with techniques like mass spectrometry, form the basis of Activity‐Based Protein Profiling (ABPP), wherein active subsets of enzymes in cell or tissue extracts are globally profiled. When applied to cell lysates, these probes form stable covalent complexes with enzymes, facilitating (i) structural characterization of probe‐enzyme interactions, (ii) proteomic profiling to uncover novel enzymes or binding partners, and (iii) high‐throughput inhibitor screening. Together, these applications provide both mechanistic insights and avenues for therapeutic discovery within the ubiquitin system. Notably, because ABPs only react with the active form of an enzyme (for example, a DUB with a reduced catalytic cysteine), they report on the regulated activation state of enzymes in cells, not just their presence. For instance, oxidative stress (H_2_O_2_ treatment) can abrogate labeling of certain DUBs by Ub‐based probes due to cysteine oxidation, while T‐cell stimulation increased labeling of USP9X by a Ub‐ABP without changing USP9X protein levels, reflecting activation by phosphorylation.

### 2.2 Types of Warheads

A variety of electrophilic warheads have been developed to target the active‐site cysteine of Ub/Ubl enzymes. These warheads differ in reactivity, stability, and the mechanism by which they capture the enzyme. The major classes include:

#### 2.2.1 Halomethyl Ketones

These are small‐molecule probes based on chloromethyl ketone (CMK), a 4‐chloroacetylpyrrole derivative, and glycine fluoromethyl ketone (FMK) [20, 21]. These react by alkylating the cysteine via an SN_2_ displacement. CMK probes target multiple UCH and USP DUBs, whereas FMK is effective against SUMO‐specific proteases (SENPs). They are highly reactive but relatively unstable. Acyloxymethyl ketones (AOMKs), often used in broad‐spectrum cysteine protease probes, were explored for DUB probes [22, 23]. Consistent with its high reactivity toward Cys nucleophiles, the AOMK HA‐UbTF_3_BOK labeled many E1, E2, and E3 enzymes in addition to DUBs. AOMKs are more reactive than vinyl sulfones but showed similar DUB labeling in cell lysates, likely due to poor stability. Because of potential off‐target reactivity and instability, halide‐based warheads are less common in modern Ub/Ubl ABPs.

#### 2.2.2 Michael Acceptors (α,β‐Unsaturated Carbonyls)

These include vinyl sulfones (VS), and vinyl (methyl) esters (VME) attached to the Ub *C‐*terminus. Pioneered by Ploegh and colleagues, the first irreversible DUB probes had a *C*‐terminal vinyl methyl sulfone replacing the native Gly76, yielding Ub‐VS [24]. Ub‐VS probes covalently modify DUB active‐site cysteines via Michael addition and were shown to label DUBs in cell extracts irreversibly. Vinyl methyl ester (VME) warheads operate similarly. Interestingly, Ub‐VME was found to be slightly more reactive than Ub‐VS for certain DUBs. VS and VME probes are in widespread use and are commercially available (e.g., HA‐Ub‐VS, Biotin‐Ub‐VME) as broad‐spectrum DUB reagents. Although Michael addition is in principle reversible under strongly basic conditions, the adducts formed by Ub‐VS/VME are stable under physiological and SDS‐PAGE conditions.

#### 2.2.3 Propargyl‐Based Electrophiles

Propargyl amine (PA) warhead (also called acetylenic probe) at the *C*‐terminus of Ub/Ubl features an electrophilic triple bond. A Ub‐propargylamine (Ub‐PA) probe reacts with cysteine proteases via a unique mechanism where the cysteine attacks the β‐carbon of the alkyne, resulting in a vinyl thioether adduct. The formed thioether is stable to denaturing and reducing conditions, but notably can be cleaved under acidic conditions [25, 26]. This acid‐cleavability is advantageous for proteomic analysis: labeled enzymes can be enriched (via a biotin or alkyne tag on the probe) and then released from the probe by mild acid, simplifying mass spectrometry identification. PA probes have become valuable for profiling cysteine proteases in situ, and one study showed that incorporating a propargyl warhead in a fluorescent SUMO probe enabled imaging of SENP activities in cells. Propargyl probes tend to have high reactivity and have been observed to label a broad set of DUB families, including those that were less reactive with VS/VME (e.g., certain OTU and MJD family DUBs). Indeed, proteomic studies indicate propargyl probes can even capture the Machado‐Joseph disease DUBs (Josephins) that may be under‐represented by other probes [10, 27, 28].

#### 2.2.4 Haloalkyl Electrophiles

Electrophilic reactive groups such as chloroethylamine (Ub‐Cl) and bromoethylamine (Ub‐Br) have been introduced at the *C*‐terminus of ubiquitin to diversify the reactivity landscape of ABPs [29]. These warheads act through nucleophilic substitution mechanisms, wherein the catalytic cysteine thiol of a deubiquitinase (DUB) displaces the halogen atom, forming a stable covalent thioether adduct. Compared to Michael addition (e.g., vinyl sulfones) or thiol‐alkyne chemistry (e.g., propargylamine), these haloalkyl probes offer distinct reactivity profiles that can trap enzymes not efficiently captured by other electrophiles. Ub‐Cl and Ub‐Br derivatives have been used to investigate the active‐site environment of DUBs and related isopeptidases, particularly to assess the tolerance of different enzymes toward steric and electronic variations in warhead chemistry. Although less widely applied than propargyl‐ or vinyl‐based probes, haloalkyl probes highlight how fine‐tuning electrophile identity can broaden the coverage of ABPs across mechanistically diverse cysteine proteases in the ubiquitin system [22, 30, 31].

#### 2.2.5 Dehydroalanine (Dha)

Dehydroalanine is an electrophilic amino acid (a vinyl amino acid) that can be site‐specifically installed at the *C*‐terminus of Ub/Ubl via chemical synthesis. Dha warheads react by Michael addition of the cysteine thiolate to the dehydroalanine, forming a stable thioether. A key example is Ub‐Dha, a probe in which the *C*‐terminal Gly of ubiquitin is replaced by dehydroalanine. Ub‐Dha has been shown to traverse the E1‐E2‐E3 cascade like native ubiquitin (via thioester transfer), but at each step, the transient thioester intermediate can be converted into a permanent covalent linkage if the Dha reacts with the active‐site cysteine [32]. This “cascading probe” behavior allows trapping of active E1s, E2s, or E3s in a context‐dependent manner. Dha‐based probes for Ubls have also been prepared; for instance, a fluorescent UFM1‐Dha probe labeled the UFM1 E1 enzyme UBA5 (but not the Ub E1) in cells and could profile UFM1 transfer activity [33]. Overall, Dha provides a versatile warhead with enzymatic compatibility (mimicking a Cys or Ser intermediate) and high reactivity.

#### 2.2.6 Azapeptide Esters

Ubiquitin probes featuring azapeptide ester at the Ub *C*‐terminus have recently emerged as a next‐generation strategy for profiling DUBs and other cysteine enzymes in the ubiquitin system. In these probes, the terminal Gly residue of ubiquitin is replaced with an azaglycine moiety, in which the Gly α‐carbon is substituted by a nitrogen. This design yields a *C*‐terminal O‐acyl hydrazine (azapeptide ester) functionality, making essentially a ubiquitin acyl‐hydrazide capped with an ester group [34]. The general structure can be represented as Ub‐CO‐NH‐NH‐CO_2_R (where R is an alkyl or aryl group), replacing the normal ‐CO‐CH_2_‐CO_2_H of Gly76. This modification more closely mimics the native peptide linkage of a natural isopeptide‐bonded ubiquitin conjugate, both in chemical geometry and in electronic character. Conventional ubiquitin‐based probes are structurally different from native ubiquitinated protein. Their use in activity‐based profiling may fail to capture subsets of cysteine enzymes that depend on stricter substrate engagement or whose active sites cannot accommodate the transition‐state mimics generated by standard probe designs. An azapeptide ester is structurally highly similar and therefore maintains a similar binding pattern to a target enzyme.  As a result, azapeptide ester warheads preserve native‐Ub‐protein features for substrate recognition by cysteine enzymes: the ubiquitin's *C*‐terminus can engage the enzyme's binding pocket similarly to a genuine substrate, rather than presenting a highly distorted electrophile as in traditional probes.

Mechanistically, azapeptide esters react with cysteine proteases in the same manner as the physiological ubiquitin‐protein cleavage (i.e., via an acyl transfer reaction). The DUB's active‐site cysteine performs a nucleophilic attack on the warhead's carbonyl (analogous to the scissile bond in a ubiquitinated protein), forming a tetrahedral intermediate that collapses with expulsion of the leaving group (the ester's alkoxy fragment). The enzyme becomes covalently linked to ubiquitin via a thiocarbamate bond, transiently mimicking the catalytic acyl‐enzyme intermediate of normal ubiquitin hydrolysis. Notably, this contrasts with older warhead chemistries: for example, ubiquitin vinyl methyl ester (Ub‐VME) and vinyl sulfones form irreversible thioether adducts by Michael addition, while Ub‐PA undergoes an unusual 1,2‐addition to yield a vinyl thioether. Those reactions, although effective in trapping DUBs, do not occur according to normal enzymatic reactions. In contrast, azapeptide ester probes induce the same kind of acylation of the catalytic cysteine seen in bona fide ubiquitin processing.

#### 2.2.7 Other Warheads

Additional electrophiles have also been explored. Vinyl amides (VA) have been used especially in di‐Ub or poly‐Ubl probes to create crosslinking mimics of isopeptide bonds. For example, a diSUMO‐2 probe with a vinyl amide linkage at the junction was able to covalently trap SUMO‐chain‐specific proteases [35]. Other specialized warheads include aziridines or epoxides (small ring electrophiles) and photo‐reactive groups [36, 37]. Each warhead brings trade‐offs in reactivity vs. stability and selectivity. For instance, an overly reactive probe can sometimes modify off‐target cysteine residues on a protein. One notable case: HA‐Ub‐VS was found to react with a non‐catalytic cysteine on OTUB1, a DUB, leading to a mislabeled band and underscoring that probe labeling must be interpreted with caution [38]. Table 1 summarizes the electrophilic groups used for Ub probes.

**TABLE 1:** Cysteine reactive electrophilic group employed in various mono Ub/UbI probes.

Reaction mechanism	Probe name	Structure	Electrophilic group
*Nucleophilic Acyl Substitution*	Ub‐MTC	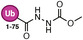	Methyl Hydrazine Carboxylate
	Ub‐ETC	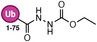	Ethyl Hydrazine Carboxylate
	Ub‐TFEGHC	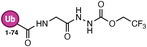	2,2,2‐trifluoroethyl‐2‐glycylhydrazine‐1‐carboxylate
*Direct (1,2) Addition*	Ub‐Al	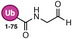	Aldehyde
	Ub‐CN	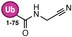	Nitrile
	Ub‐PA	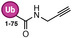	Propargylamine
*Nucleophilic Substitution*	Ub‐Cl	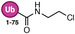	Chloroethylamine
	Ub‐Br2	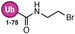	Bromoethylamine
	Ub‐Br3	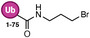	Bromopropylamine
	Ub‐TF_3_BOK	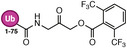	Glycine‐2,6‐trifluoromethyl Benzyloxymethyl Ketone
	Gly‐FMK	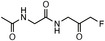	Glycine Fluoromethyl Ketone
	Ub‐Lac	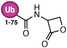	α‐Amino‐β‐lactone
	CMK	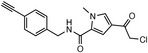	Chloromethyl Ketone
*Michael Addition*	Ub‐VS	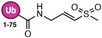	Vinyl Sulfone
	Ub‐OEtVs	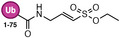	Vinyl Ethoxy Sulfone
	Ub‐VSPh	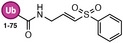	Vinyl Phenyl Sulfone
	Ub‐VME	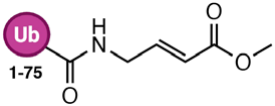	Vinyl Methyl Ester
	Ub‐VCN	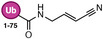	Vinyl Cyanide
	Ub‐CN	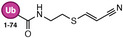	Thioacrylo Nitrile
	Ub‐Dha	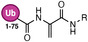	Dehydroalanine

### 2.3 Reporter Tags and Delivery

The reporter tag on an ABP is crucial for detecting labeled enzymes, and its design often depends on the experimental context. Common tagging strategies include:

#### 2.3.1 Direct Labels

Fluorophores (e.g., TAMRA, rhodamine) attached to the probe enable one‐step detection of labeled proteins by in‐gel fluorescence. For example, Ub or Ubl probes have been made with *N*‐terminal TAMRA, allowing rapid visualization of DUB or SENP activity in gels [39, 40]. The advantage is sensitivity and real‐time detection, though adding a bulky dye can reduce cell permeability and may influence binding.

#### 2.3.2 Affinity Tags

Biotinylated probes (or probes with small epitope tags like HA or FLAG) allow sensitive detection by blotting and enrichment of labeled proteins via streptavidin or antibody pulldowns [24, 34]. HA‐tagged Ub‐VS was used in the earliest studies to detect DUB labeling on Western blots. Biotin tags are highly useful for mass spectrometry workflows, where biotin‐streptavidin enrichment of probe‐labeled proteins from cell lysates can isolate low‐abundance enzymes for identification [39, 41, 42]. A caveat is that a large tag can sterically hinder the probe's access to some enzymes or cellular compartments.

#### 2.3.3 Bioorthogonal Handles

To mitigate the issues of bulky tags, many probes incorporate a small chemical handle (most often an alkyne or azide), and use click chemistry for secondary labeling [43, 44, 45]. In this two‐step strategy, the probe is incubated with the sample to label enzymes, and then a fluorescent or biotin azide is clicked on post hoc. This yields a labeled enzyme with the full reporter attached only after the reaction has taken place, minimizing interference with the enzyme targeting. Statsyuk et al. employed an alkyne tag on the adenine ring of their E1‐targeting ABP to allow subsequent fluorescent detection [46]. This two‐step labeling is now a standard approach in ABPP experiments.

Delivering ABPs into living cells is another important consideration. Protein‐based probes like full‐length ubiquitin derivatives (∼8.5 kDa) generally do not freely cross cell membranes. These are typically applied to cell lysates or permeabilized cells [17]. In some cases, microinjection or cell‐permeabilization techniques (e.g., Streptolysin‐O pores) have been used to introduce Ub probes into cells to label DUBs in situ [47, 48]. An alternative is chemical transduction: for example, a cell‐permeable probe can be made by capping a charged peptide with a cell‐penetrating peptide or using a bio‐reversible protection on acidic residues. More commonly, investigators use electroporation or similar methods to deliver probes. One study electroporated a fluorescent UFM1‐PA probe into HeLa cells and successfully visualized nuclear vs. cytosolic pools of active UFM1 protease UFSP1 [33].

Small‐molecule ABPs offer another solution to live‐cell profiling. A notable example is ABP1, a ∼500 Da molecule designed to target E1 enzymes [46]. ABP1 is inherently cell‐permeable and has been used to label E1 activities inside intact cells. Similarly, photo‐crosslinking probes for RING E3s consist of recombinant proteins (E2∼Ub conjugates) that can be added to cell extracts, or even introduced into cell‐based assays, to report on E3 activation [49]. While true live‐cell delivery of large ABPs (like ubiquitin‐based probes) remains challenging, the use of ex vivo labeling (treating cell lysates) is very informative and has been widely adopted. In summary, ABPs can be deployed in diverse formats, from in vitro biochemical assays to cell lysate profiling to, in special cases, intracellular labeling, by appropriate choice of tags and delivery methods.

## 3 ABPs for Deubiquitinases (DUBs): Ubiquitin‐Based Probes

**FIGURE 3:**
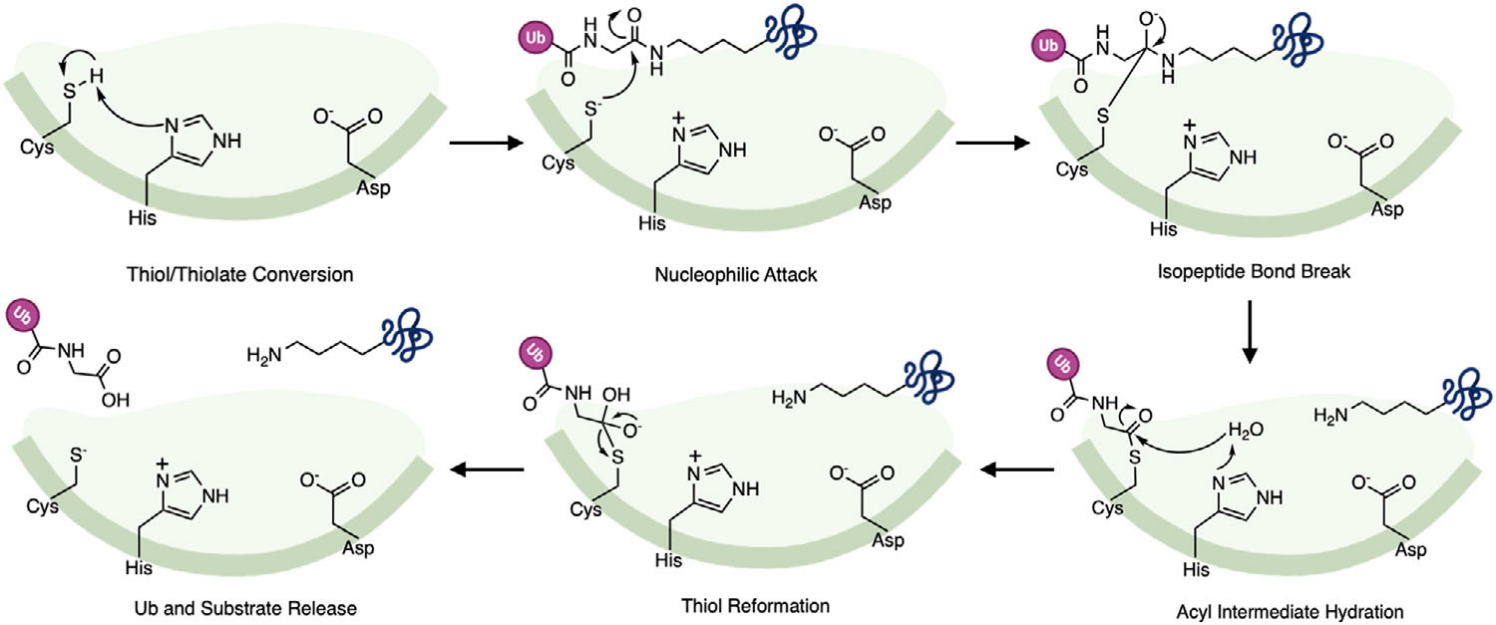
Catalytic cycle of cysteine‐based DUBs. The reaction begins with thiol/thiolate conversion, where the catalytic cysteine is activated by polarization of the adjacent histidine and stabilized by an asparagine/aspartate residue. The active thiolate then performs a nucleophilic attack on the isopeptide bond linking ubiquitin to its substrate, generating a covalent thioester intermediate. This is followed by isopeptide bond cleavage and stabilization of the acyl‐enzyme intermediate. Subsequent hydrolysis of the acyl intermediate leads to substrate and ubiquitin release, while the cysteine is regenerated through thiol reformation, resetting the DUB for another catalytic cycle.

The activity of cysteine based DUBs depends on whether the catalytic cysteine is in an inactive thiol (‐SH) or active thiolate (‐S^−^) state [50]. As shown in Figure 3, transitions between these states are triggered by substrate binding, protein complex formation, or DUB post‐translational modifications. Conformational shifts polarize the catalytic histidine often aided by an asparagine or aspartate, which lowers the cysteine pKa, promoting thiol deprotonation and thiolate stabilization [51]. The active thiolate then performs a nucleophilic attack on the isopeptide bond of ubiquitin‐ protein or ubiquitin chains, generating a thioester intermediate before hydrolysis releases the ubiquitin [52, 53]. This resets the cysteine to the thiolate state, allowing the DUB to initiate a new catalytic cycle.

**FIGURE 4:**
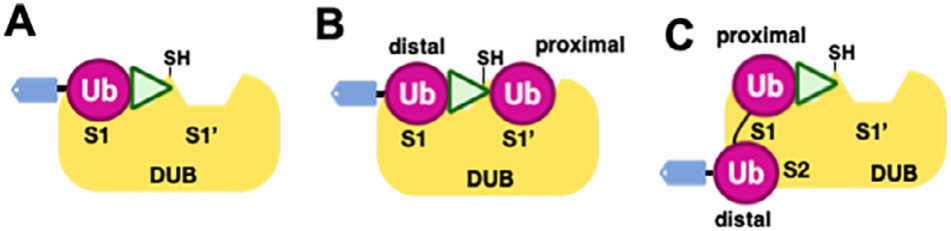
Recognition of substrate in DUBs. (A) A monoubiquitin‐based activity probe bearing a *C*‐terminal electrophilic warhead engages the catalytic cysteine in the DUB active site. Recognition is primarily mediated through interactions between ubiquitin and the canonical S1 ubiquitin‐binding site, which positions the *C*‐terminus of ubiquitin for nucleophilic attack. (B) A diubiquitin‐based probe provides additional recognition by simultaneously engaging the S1 site (proximal ubiquitin) and the S1′ site (distal ubiquitin), thereby increasing the binding affinity and enabling the linkage‐specific discrimination. (C) Certain DUBs contain extended ubiquitin‐binding surfaces, including S2 sites, which accommodate additional ubiquitin moieties from longer polyubiquitin chains. Engagement of S1, S1′, and S2 sites enables selective recognition of specific chain lengths and topologies.

Activity‐based probes targeting deubiquitinases were the first to be developed in the Ub field and remain the most extensively used. The majority of DUBs are cysteine proteases (USP, UCH, OTU, MJD, ZUP1, and MINDY family DUBs), except for the JAMM metalloprotease family, which requires a different approach (JAMM DUBs are not directly targetable by cysteine‐reactive ABPs). As shown in Figure 4, DUB ABPs typically exploit full‐length ubiquitin as the recognition element, capitalizing on ubiquitin's ability to bind the conserved “S1” site of DUBs (the site that accommodates the distal Ub of a cleavable chain or Ub‐protein conjugate). By installing an electrophilic trap at Ub's *C*‐terminus, these probes covalently modify the catalytic cysteine during what would be the cleavage reaction. Although monoUb/Ubl probes are widely applied, more sophisticated designs have emerged to interrogate additional substrate‐binding sites of DUBs/ULPs. By incorporating an internal electrophile between two Ub units in a diUb chain, these probes engage both S1 and S1′ sites, enabling covalent capture of the catalytic cysteine and revealing linkage preferences [54]. Likewise, non‐hydrolyzable diUb probes with *C*‐terminal warheads probe the S2 site contribution to specificity [27]. Thus, tailoring the recognition element and strategically placing the warhead allows the design of ABPs that selectively interrogate distinct DUB/ULP activities.

**FIGURE 5:**
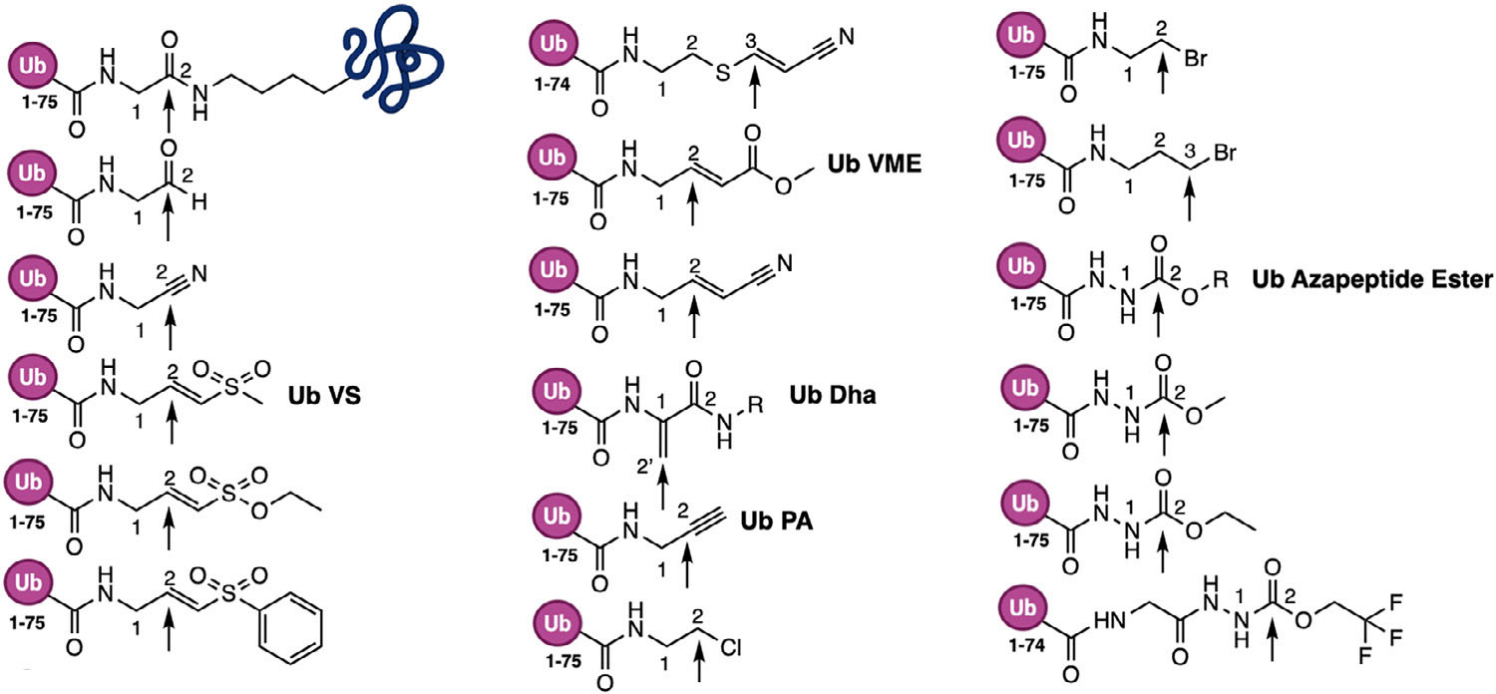
Ubiquitin based activity probes. Ubiquitin activity‐based probes developed to study the cysteine enzymes in the ubiquitin signaling cascade. A model of a ubiquitinated substrate is shown for comparison, with the Cα and carboxyl atom of ubiquitin's terminal Gly76 labeled as position 1 and 2 respectively. The arrow at position 2 in the Ub‐sub complex marks the scissile bond cleaved by DUBs, forming a transient thioester with the catalytic cysteine. Arrows in various Ub based probes denote the electrophilic sites designed to covalently trap active‐site cysteine of the enzymes across ubiquitin/deubiquitination pathway (Adapted with permission from Ref. [34], 2025, J. Am. Chem. Soc.).

The warhead at the Ub *C*‐terminus is the key determinant of ABP reactivity and selectivity. These groups are electrophiles designed to covalently trap the catalytic cysteine of DUBs [55]. Different chemistries enable distinct mechanisms: direct addition (e.g., aldehyde, nitrile), Michael addition (e.g., vinyl sulfone), or nucleophilic substitution (e.g., bromo‐ or chloroalkyl derivatives). Photoreactive moieties have also been used to crosslink nearby residues upon UV exposure [56]. Overall, warhead chemistry combined with Ub recognition element governs probe selectivity and labeling efficiency. Figure 5 represents the ubiquitin activity‐based probes that have been developed over the years.

First‐generation DUB ABPs were monoubiquitin probes with simple *C*‐terminal warheads. The earliest ubiquitin probe developed was ubiquitin aldehyde (UbAl), which acts as a potent inhibitor of cysteine enzymes in the ubiquitin cascade [57, 58]. It forms a stable thiohemiacetal adduct via 1,2‐direct addition with the catalytic cysteine, thereby covalently trapping the enzyme and blocking its activity. Later, ubiquitin nitrile (Ub‐CN) was developed as another probe, functioning as a potent inhibitor of the 26S proteasome isopeptidase by forming a thioimidate adduct with the catalytic cysteine [59]. However, like UbAl, the resulting covalent enzyme‐probe complexes were unstable in aqueous solution and underwent hydrolysis, limiting their utility. For this reason, a series of ubiquitin probes that contain a Michael acceptor or an alkyl halide were synthesized. As noted, the landmark work by Borodovsky et al. introduced a vinyl sulfone at Ub's *C*‐terminus, creating the first irreversible DUB probe (Ub‐VS) [24]. This probe, when appended with an epitope tag (HA‐Ub‐VS), irreversibly attached to active UCH and USP DUBs in cell lysates and produced an observable gel shift for labeled enzymes. The advent of Ub‐VS overcame the earlier limitation by yielding a stable thioether linkage, compatible with harsh electrophoresis conditions. Shortly after Ub‐VS, related Ubl‐VS probes were reported for several ubiquitin‐like proteins (NEDD8, ISG15, SUMO‐1) and even for the autophagy Ubls (LC3/GATE‐16/GABARAP) demonstrating the broad applicability of the vinyl sulfone warhead in targeting Ubl‐specific proteases [60].

In 2013, Ovaa and colleagues unexpectedly discovered that propargylamine (Prg, also referred to as PA), though generally considered chemically inert, becomes highly reactive when positioned at the *C*‐terminus of ubiquitin (Ub‐PA) [61]. In this context, the terminal alkyne undergoes covalent capture by the catalytic cysteine of DUBs, while showing little to no reactivity toward unrelated cysteine proteases. The reaction mechanism was initially attributed to a proximity‐driven thiol‐alkyne addition, involving direct nucleophilic attack by the catalytic thiolate [61, 62]. More recent studies confirmed the alkyne intermediate as the key reactive species, although the precise contribution of cysteine proximity to the reaction remains unresolved. Importantly, Ub‐PA labels all major classes of the cysteine DUBs, establishing propargylamine as a highly versatile and widely adopted warhead for Ub/Ubl ABPs [63].

Over the past two decades, many DUB probes have been developed, differing in warheads and configurations. Ub‐VME and Ub‐PA probes, for instance, became widely used alternatives with improved reactivity or specialized uses as described. These monomeric Ub probes target the DUB's S1 site and are generally effective against a broad spectrum of DUBs (especially USPs and UCHs). Certain DUB families, however, posed challenges. OTU‐family DUBs, for example, can exhibit narrower active‐site clefts, precise geometric alignment, stringent substrate preferences, and sometimes show weak reactivity with the probes mentioned earlier. These conventional ubiquitin probes differ structurally from native ubiquitinated proteins and often react with catalytic cysteines through mechanisms unrelated to the enzymes’ physiological chemistry. Consequently, when used in activity‐based protein profiling, these probes may fail to capture cysteine enzymes in the ubiquitination/deubiquitination cascade that demand strict substrate recognition or whose active sites are incompatible with the intermediates formed by such probes.

**FIGURE 6:**
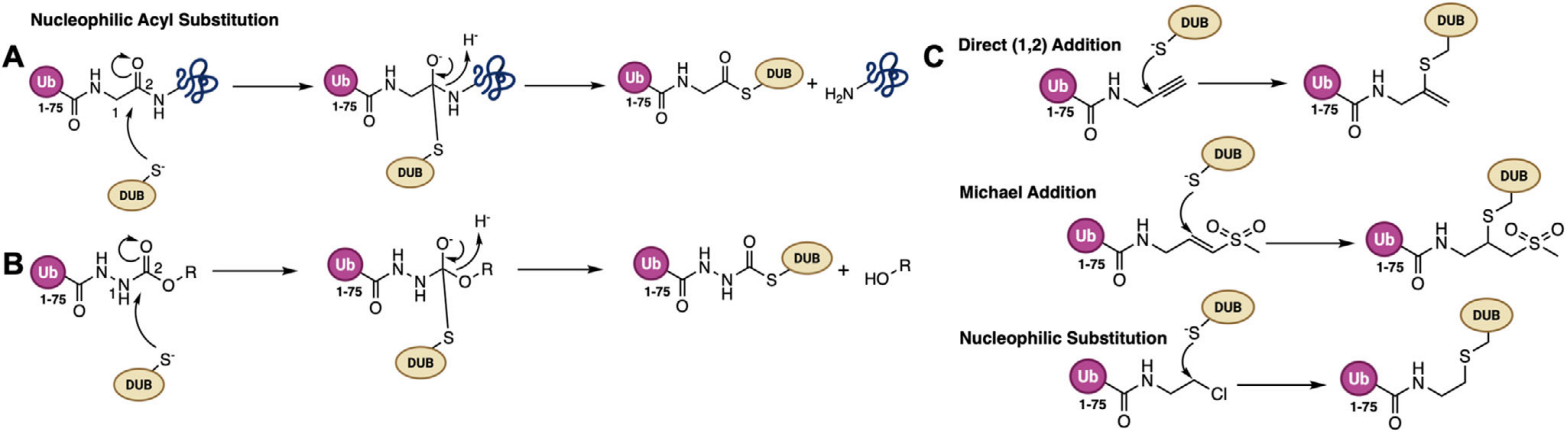
Mechanistic basis of DUB catalysis and covalent probe engagement. (A) Canonical mechanism of DUB‐mediated isopeptide bond hydrolysis. In a nucleophilic acyl substitution reaction, the catalytic cysteine attacks the *C*‐terminal carbonyl of ubiquitin, forming a thioester intermediate, followed by amine release and hydrolysis to regenerate free Ub. (B) Ubiquitin azapeptide ester probes, that react with the DUB active‐site cysteine in a manner analogous to natural ubiquitinated substrates, forming a tetrahedral transition state characteristic of acyl transfer reactions, wherein the resulting oxyanion is stabilized by the enzyme's anion hole (Adapted with permission from Ref. [34], 2025, J. Am. Chem. Soc.). (C) Probes that react with the catalytic cysteine different from native enzymatic catalysis. These reactions can be categorized into direct (1,2) addition (e.g., Ub‐PA), Michael addition (e.g., Ub‐VS), and nucleophilic displacement (e.g., Ub‐Cl), depending on the nature of the electrophiles.

To overcome that limitation and further refine probe design and target coverage, a new class of ubiquitin‐based ABPs, ubiquitin azapeptide ester probes, was introduced recently as a next‐generation tool [34]. These probes incorporate an azaglycine residue at ubiquitin's *C*‐terminus (in place of Gly76), yielding a warhead that almost resembles a natural isopeptide linkage. Upon reaction with a DUB's active‐site cysteine, an Ub azapeptide ester probe undergoes the same nucleophilic acyl substitution (Figure 6A) as a genuine ubiquitinated substrate, forming a tetrahedral intermediate and resulting in a stable enzyme‐Ub thiocarbamate adduct. As shown in Figure 6B, an ubiquitin azapeptide ester will react with a DUB active site cysteine similarly as a ubiquitinated protein substrate to form a tetrahedral transition state, typical for acyl transfer reactions, that has an oxygen anion stabilized by the anion hole in the enzyme [64]. In contrast to conventional electrophilic traps (e.g., VS or PA) that engage cysteines via non‐native (Figure 6C) Michael and direct (1,2) addition or nucleophilic substitution (Ub‐Cl) the azapeptide's electrophilic carbon is part of a peptide backbone (a carbamate) and is intrinsically more electron‐deficient, which enhances its reactivity toward nucleophiles. By offering a more native‐like binding and transition state, ubiquitin azapeptide esters can better accommodate strict substrate‐binding requirements of certain DUBs (such as OTU domains) and label any cysteine‐based ubiquitin enzymes including E1, E2, and E3 ligases by mimicking their thioester‐linked Ub substrates.

In practice, ubiquitin azapeptide esters have indeed demonstrated broad reactivity and enhanced sensitivity. In side‐by‐side comparisons, these probes labeled more active enzymes and produced stronger signals than a traditional propargyl probe (Ub‐PA) in both cell and tissue lysates. In a panel of DUBs tested, each azapeptide probe showed reactivity comparable to or greater and quicker than Ub‐PA, and several OTU‐family DUBs that showed little or no labeling with Ub‐PA (e.g., OTULIN, OTUD2, OTUD7C) were efficiently captured by azapeptide probes. Especially noteworthy was the Josephin‐family DUB Ataxin‐3: in the human cell lysate, this polyubiquitin chain‐preferring enzyme was barely modified by Ub‐PA but was readily tagged by ubiquitin azapeptide esters. Furthermore, because they form covalent adducts via a near‐native mechanism, Ub azapeptide probes can target upstream ubiquitin‐handling enzymes as well including E1, E2, and cysteine‐based E3 ligases. This potential was confirmed by direct labeling of representative E1, E2, and E3 enzymes in vitro. Thus, ubiquitin azapeptide esters have emerged as powerful next‐generation ABPs, combining superior reactivity with authentic substrate mimicry to expand the detectable landscape of active ubiquitin‐system enzymes including those that previously escaped conventional probes. Their use has even revealed distinct DUB activity profiles in different mouse tissues, underscoring the potential of these probes in mapping context‐specific enzyme function and disease‐associated dysregulation.

In addition to profiling known DUBs, Ub‐based ABPs have been instrumental in discovering new DUB enzymes and functions. Early chemoproteomic applications of radiolabeled probes (e.g., ^125^I‐Ub‐VS or ^125^I‐Ub‐Al) in cell extracts led to the identification of novel DUBs by comparing wild‐type vs. DUB‐knockout lysates. For instance, labeling of yeast lysates with Ub‐Al followed by purification and mass spectrometry helped isolate a new DUB (subsequently identified as the yeast USP14 homolog) as a proteasome‐associated isopeptidase [65]. Similarly, ABPs have helped assign DUBs to specific complexes or organelles. The permanent nature of the enzyme‐probe adduct allows stringent washes and tandem affinity purifications to fish out DUB‐containing complexes. A recent advance introduced a metalloDUBprobe in which ubiquitin was engineered with a Zn^2^^+^‐chelating group at its *C*‐terminus. This design expands the scope of ABP chemistry and points toward the development of new, more potent tools for targeting metalloprotease‐type deubiquitinases [66].

**FIGURE 7:**
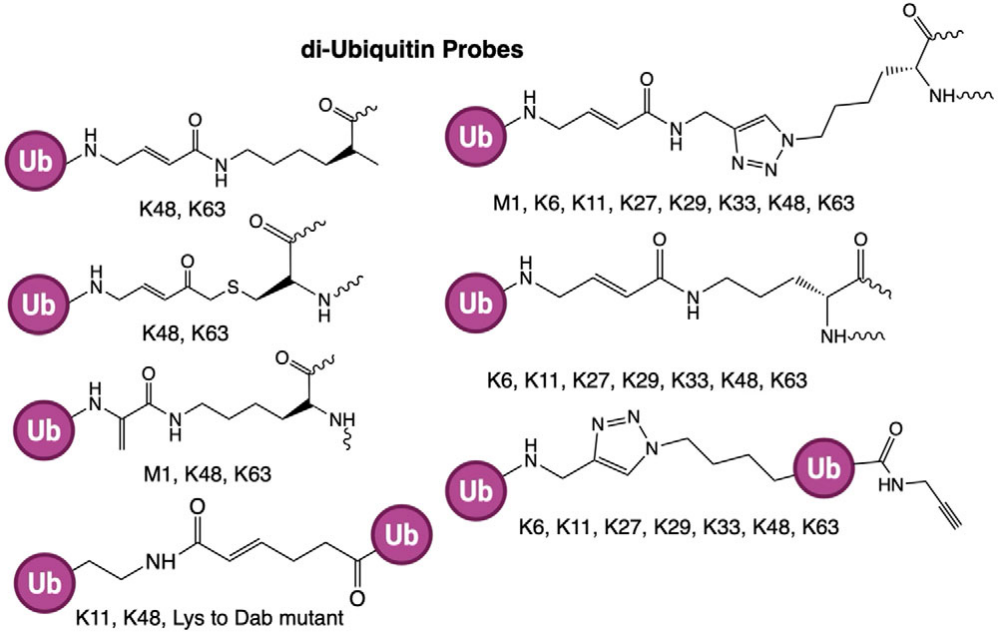
di‐Ubiquitin based probes. di‐ubiquitin probes consist of two Ub moieties linked through a defined isopeptide bond (e.g., K11, K48, K63, M1, or other Lys‐linkages) to mimic the native chain topology. K48‐diUb probes identify DUBs that target proteasomal degradation signals. K63‐diUb probes reveal enzymes acting in signaling and DNA repair pathways. M1‐linear diUb probes capture DUBs like OTULIN that process Met1 linkages.

Beyond broad‐spectrum probes, several other DUB probes have incorporated more complex recognition elements to confer linkage or substrate specificity. Many DUBs recognize not only the single Ub at the scissile bond, but also adjacent Ubs in a chain (through an S2 site) or the substrate protein on the proximal side (through an S1’ site). To profile these preferences, researchers developed di‐ubiquitin (diUb) ABPs that present two ubiquitin units joined via a defined isopeptide linkage, with a warhead built into the linkage or at the distal Ub's *C*‐terminus. These diUb probes can selectively label DUBs that prefer certain chain linkages or that require an extended chain context for optimal binding. For example, Iphöfer et al. prepared Ub‐K48/K63 peptide‐isopeptide probes (diUb mimetics) and found that certain DUBs (such as the K63‐specific AMSH and K48‐preferring OTUB1) were labeled only by the corresponding linkage probe [67]. Different designs of diUb ABPs have been reported (Figure 7): some use a non‐cleavable isopeptide isostere (e.g., a stable triazole or a thioether linkage) with a warhead at one terminus, while others incorporate an electrophile directly in the isopeptide bond to trap the enzyme during cleavage [68, 69, 70, 71]. Ovaa and co‐workers pioneered the conceptual design of diubiquitin activity‐based probes based on K11‐ and K48‐linked architectures. To preserve the native isopeptide linkage length in these probes, the lysine residue at the linkage position in the proximal ubiquitin was replaced with diaminobutyric acid (Dab), enabling installation of the reactive functionality while maintaining near‐native geometry [72]. An elegant approach by Flierman et al. used bifunctional chemistry to create “bisubstrate” probes that simultaneously occupy the S1 and S2 Ub‐binding sites. These non‐hydrolyzable di‐Ub probes revealed pronounced linkage preferences for many DUBs. For instance, OTUD2 labeled strongly with a K11‐linked ABP but not with K63, despite being able to cleave multiple chain types in vitro, highlighting how S2 interactions modulate catalysis [73]. Likewise, OTULIN (specific for linear M1 chains) did not react with an M1 diUb probe that lacked a true peptide bond, indicating that linear chains require a native peptide for recognition. Overall, diUb ABPs have become valuable for mapping DUB linkage specificities in a manner that closely mimics physiological substrates.

In parallel, substrate‐based ABPs have been devised to target DUBs that act on ubiquitinated protein substrates. One innovative example is an activity‐based probe that uses a ubiquitinated protein as the recognition element: a semisynthetic probe consisting of Ub linked to a fragment of PCNA was developed and (a substrate monoubiquitinated in DNA repair) terminated with a warhead. This probe specifically fished out USP1 (the DUB for Ub‐PCNA) from cell extracts, demonstrating how incorporating substrate context can confer selectivity to an ABP [74]. Similarly, researchers have used peptide‐based probes corresponding to the *C*‐terminus of Ub or Ubls to target specific proteases: short peptides (e.g. the *C*‐terminal 4–6 residues of SUMO or NEDD8 with a VS warhead) can sometimes suffice for enzymes like SENP or NEDP1which have smaller binding pockets than DUBs. However, in general, full‐length Ub/Ubl provides far higher affinity and selectivity, and thus most probes utilize the entire ubiquitin fold. In a major conceptual advance in the substrate‐based activity probes, Meledin et al. reported the semisynthetic Ub‐Dha‐globin probe, in which ubiquitin bearing a dehydroalanine (Dha) electrophile was site‐specifically conjugated to α‐globin [75]. This probe preserves the full protein substrate context while presenting a reactive warhead at the isopeptide junction, enabling covalent capture of DUBs such as USP15 that recognize globin‐linked ubiquitin. These activities are not efficiently profiled by monomeric Ub probes. Using this strategy, the authors identified DUB activities with potential roles in globin regulation. Shortly thereafter, Jbara et al. extended this concept to chromatin biology by developing Ub‐Dha‐H2A, a histone‐based substrate probe in which Ub‐Dha was conjugated to histone H2A [76]. This probe selectively engaged histone‐directed DUBs and enabled activity‐based profiling of enzymes involved in deubiquitination of nucleosomal substrates. Together, these studies established UbDha‐protein conjugates as a powerful class of ABPs that combine irreversible covalent trapping with authentic substrate recognition, allowing interrogation of substrate‐ and context‐dependent DUB activities that are inaccessible using conventional monoUb‐based probes.

Importantly, DUB ABPs not only identify enzymes but also allow assessment of their regulation and inhibitor engagement. By comparing ABP labeling with and without treatment, one can infer the activation state of DUBs. For example, as noted, oxidation of the catalytic cysteine by peroxide reduces probe labeling and phosphorylation‐based activation can increase labeling. ABPs have been used to verify target engagement of DUB inhibitors: if a small‐molecule inhibitor occupies the active site, it will block ABP labeling. This competitive ABPP format was used to characterize, for instance, the selectivity of USP7 inhibitors by showing loss of HA‐Ub‐VME labeling on USP7 in inhibitor‐treated cell extracts. Thus, ABPs are valuable in drug discovery for the ubiquitin system.

In summary, ubiquitin‐based ABPs have greatly advanced our ability to profile DUB activity. Probes like HA‐Ub‐VS, Biotin‐Ub‐VME, and Cy5‐Ub‐PA are now standard tools in ubiquitin research laboratories worldwide. They enable the enrichment and characterization of known DUBs, the discovery of novel DUBs or DUB modulators, and the dissection of DUB specificity in terms of linkage and context. These probes set the stage for expanding the ABP concept to the myriad proteases of ubiquitin‐like modifiers, as discussed next.

## 4 ABPs for Ubiquitin‐Like Protein Specific Proteases (ULPs)

**FIGURE 8:**
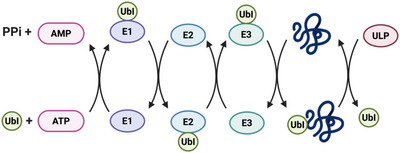
Ubiquitin‐like proteins (UbI) conjugation/deconjugation cascade. Similar to ubiquitin, Ubiquitin‐like proteins (UbI) are conjugated to substrate lysines through a conserved E1‐E2‐E3 enzymatic cascade. UbI modifications are also reversible and dynamically regulated by ubiquitin‐like proteins specific proteases (ULPs).

**FIGURE 9:**
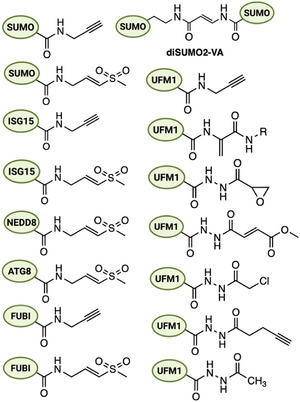
UbI activity‐based probes. Activity‐based probes for different UbIs to target the ULPs of the respective pathways.

Ubiquitin‐like modifiers (Ubls) such as SUMO, NEDD8, ISG15, FAT10, UFM1, URM1, and the autophagy‐related ATG8/ATG12 families each have dedicated proteases responsible for processing Ubl precursors and removing Ubls from substrates (Figure 8). Given the parallels to ubiquitin, it is natural that similar ABPs have been developed (Figure 9) for many Ubl‐specific proteases (also called ULPs, ubiquitin‐like proteases). In many cases, the same warheads used for Ub have been transplanted to Ubl scaffolds. Indeed, the earliest Ubl ABPs were the VS probes for NEDD8, SUMO‐1, and ISG15 prepared by Hemelaar et al.in 2004. Below, we survey ABPs for each Ubl class, noting unique challenges and insights gained from these tools.

### 4.1 SUMO

SUMO (Small Ubiquitin‐like Modifier) is processed and deconjugated by a family of SENP proteases (SENP1‐3,5‐7 in humans) as well as a few other enzymes (USPL1, DeSI1‐2) that have SUMO‐specific isopeptidase activity [77]. Most SENPs are cysteine proteases, making them amenable to ABP targeting. Full‐length SUMO‐based probes with vinyl sulfone or propargyl warheads have been key in profiling SENP activity [23, 35]. For example, HA‐tagged SUMO‐1‐VS and SUMO‐2‐VS probes have been used to label active SENPs in cell extracts [78]. These revealed distinct preferences: SENP1 and SENP2 (broadly acting SENPs) readily labeled with both SUMO1 and SUMO2/3‐derived probes, whereas SENP3 and SENP7 (polySUMO chain proteases) preferentially reacted with SUMO‐2‐based probes. In one study, Ovaa and colleagues synthesized rhodamine‐labeled SUMO‐1, ‐2, and ‐3 propargyl probes and demonstrated in cell lysates that SENP6 and SENP7 (which primarily act on SUMO‐2/3 chains) showed little labeling by SUMO‐1‐PA but significant labeling by SUMO‐2/3‐PA [79]. This mirrors their biological specificity for SUMO‐2/3‐modified substrates. Moreover, all SUMO isoform probes labeled SENP1/2 strongly, consistent with those enzymes’ broad specificity. The Melchior lab employed HA‐SUMO‐VME probes in HeLa cell lysates, combining pull‐downs with mass spectrometry to profile SUMO proteases. Sequential enrichment with SUMO1‐VME and SUMO3‐VME from large‐scale cultures enabled the discovery of a previously unknown SUMO protease, USPL1 [80].

Fluorescent SUMO ABPs have also enabled cellular imaging of SENP activity. It was also reported that cell‐permeable TAMRA‐SUMO2‐propargyl probes, when microinjected or electroporated into cells, covalently labeled endogenous SENPs, allowing direct visualization of enzyme localization and activity. Such studies found, for instance, that overexpressed SENP1 and SENP2 could relocalize SUMO‐2 probe signal to the nucleus, consistent with their nuclear functions [81]. Another sophisticated ABP involved a diSUMO vinyl amide probe designed to mimic a polySUMO chain: a nonhydrolyzable K11‐linked SUMO2 dimer (Figure 9) with a vinyl amide at the linkage was synthesized to trap chain‐specific proteases [79]. This diSUMO‐VA probe reacted with all SENPs (1,2,3,5,6,7) except SENP8 (the NEDD8‐specific enzyme), and revealed that SENP3 has a pronounced preference for cleaving SUMO2 chains over SUMO1‐modified targets [82, 83]. Thus, ABPs have been invaluable in dissecting SUMO protease specificity.

### 4.2 NEDD8

NEDD8 is a ubiquitin‐like protein that modifies cullin proteins and other substrates to regulate protein degradation pathways. The proteases that remove NEDD8 (deneddylases) include NEDP1 (also called DEN1 or SENP8) and, interestingly, two ubiquitin‐specific proteases: UCHL3 and USP21 can process NEDD8 as well as Ub [84, 85, 86]. ABPs for NEDD8 were among the first Ubl probes: NEDD8‐VS was made by similar semi‐synthesis to Ub‐VS. Early profiling with radiolabeled NEDD8‐VS identified a set of NEDD8‐reactive enzymes in cell lysates, notably NEDP1/DEN1 or SENP8 (canonical deneddylases), but also UCHL1, a neuron‐specific ubiquitin hydrolase, which was a surprising off‐target that can bind NEDD8. This finding highlighted that some ostensibly Ub‐specific proteases have dual specificity (UCHL1 can cleave NEDD8, consistent with structural homology between Ub and NEDD8). Similarly, USP21 is known to have dual Ub/NEDD8 activity, though it is a USP family member [16, 82].

While modern Ub‐ABPs are well established, equivalent NEDD8 probes can also be developed. These could be used to confirm the activity of NEDP1 in cell lysates and to detect NEDD8‐specific pools of activity. For example, treatment of cells with MLN4924 (a NEDD8 E1 inhibitor) blocks cullin neddylation, leading to loss of NEDD8‐conjugates; NEDD8‐ABPs can be applied to such samples to examine whether deneddylase activity is influenced by cullin deneddylation status. While less commonly reported than Ub or SUMO probes, NEDD8 probes are invaluable for studying the cullin‐RING ligase regulatory axis. One study using a Cy5‐NEDD8‐PA in tissue extracts found tissue‐specific patterns of active deneddylases, underscoring that these enzymes (like DEN1) may be differentially regulated across cell types [87].

### 4.3 ISG15

ISG15 is an interferon‐induced Ub‐like modifier composed of two Ub‐like domains in tandem. The primary deISGylating enzyme in humans is USP18 (also known as UBP43), a USP‐family cysteine protease dedicated to ISG15 [88]. ABPs based on ISG15 have been created to profile USP18 activity. ISG15‐VS probes were prepared by Ploegh's group and shown to label USP18 in cell lysates, with specificity—in other words, few other proteins in lysates reacted, except for some low‐level cross‐reactivity with ubiquitin proteases like USP5. That low‐level labeling of USP5 by an ISG15‐VS probe revealed USP5's capacity to bind the *C*‐terminal LRLRGG motif common to both Ub and ISG15, again illustrating overlapping specificity between Ub and Ubl pathways. Using the same probe, a screen of DUBs revealed deISGylase activity for USP2, USP13, and USP14, while an extended microarray screen with ISG15‐VME further identified USP28, and to a lesser extent USP5 and USP51, as deISGylases [89].

A refined ISG15 probe design used a propargylamine warhead and a fluorescent tag: Rhodamine‐ISG15‐PA probe which successfully trapped USP18 from human cell lysates [88, 90]. In cells overexpressing USP18, only catalytically active USP18 (and not a catalytically dead mutant) was labeled by the ISG15‐PA probe, confirming the probe's specificity for the active‐site cysteine. This probe was used to monitor USP18 activity under different stimuli. Given ISG15's role in antiviral responses, such ABPs can help screen for modulators of USP18 or for alternative ISG15 isopeptidases (though USP18 is unique in mammals, some viruses encode their own deISGylases that could be probed similarly).

### 4.4 FAT10

FAT10 is a diubiquitin‐like modifier (two Ub‐homologous domains in a single polypeptide) that targets substrates for proteasomal degradation independently of ubiquitin. Uniquely, no dedicated deconjugating enzyme for FAT10 has been identified to date [91]. Current evidence suggests that once FAT10 is attached to a substrate, both the substrate and FAT10 are rapidly degraded together by the proteasome, rather than being recycled. As a result, there is no known “FAT10 isopeptidase” to target with an ABP. Researchers have searched for de‐FAT10‐ylating activities, but none have been confirmed. Therefore, ABPs for FAT10 have not been reported in the literature, and FAT10‐VS or ‐PA probes used in exploratory studies showed no specific labeling, consistent with the absence of a dedicated protease. FAT10 precursors do undergo a *C*‐terminal processing step (removal of a diglycine motif) by an unidentified hydrolase, but it may be that this processing is autocatalytic or performed by the conjugation E1/E2 system itself. In summary, FAT10 remains an outlier where the lack of a deconjugase means the typical ABP approach has limited applicability. Any future discovery of a FAT10‐protease (for example, a contextual protease in specific tissues) could revive interest in FAT10 ABPs.

### 4.5 UFM1

UFM1 (Ubiquitin‐Fold Modifier 1) is an 83‐amino‐acid Ubl involved in ER stress and ribosome‐associated quality control. Two cysteine proteases, UFSP1 and UFSP2, remove UFM1 from substrates (UFMylation is the term for UFM1 conjugation) and also process the UFM1 precursor to reveal its C‐terminal Gly. ABPs for UFM1 have been developed recently, aided by synthetic methods. Given the relatively low conservation of UFM1 versus Ub, full‐length UFM1 probes are used for specificity. UFSP1 and UFSP2 were identified using a FLAG‐UFM1‐VME probe incubated with mouse tissue extracts, followed by immunoprecipitation and mass spectrometry [92]. While only UFSP1 was directly detected with the probe, UFSP2 was revealed through BLAST analysis and shown to bind the UFM1 ABP. Notably, humans exclusively express catalytically active UFSP2, as UFSP1 is inactive, and UFM1 probes have been crucial for elucidating the catalytic mechanism of UFSP2 [93, 94, 95]. A rhodamine‐UFM1‐propargylamine probe was chemically synthesized and shown to label UFSP1 and UFSP2, with an interesting result: UFSP1 was trapped much more efficiently than UFSP2. This likely reflects the lower activity or abundance of UFSP2 in the tested system, or differences in enzyme kinetics. By electroporating the Rho‐UFM1‐PA probe into cells, researchers visualized active UFSP1 within cells, noting its nucleolar localization and confirming it was the major active UFM1 protease in those cells. Catalytically inactive UFSP1 (Cys‐to‐Ala mutant) did not colocalize with the probe, reinforcing that labeling was activity‐dependent.

Additionally, a UFM1‐Dha probe was prepared to target the UFM1 conjugation enzyme (E1) UBA5 [33]. UFM1‐Dha reacted with UBA5's catalytic cysteine, forming a covalent UBA5‐UFM1 complex detectable on gels. Notably, this UFM1‐Dha did not cross‐react with the canonical Ub E1 (UBA1), indicating specificity for the UFM1 pathway of the UFMylation cycle: UFM1‐PA for deufmylases (UFSPs) and UFM1‐Dha (or other electrophiles) for conjugation enzymes [96]. Given UFM1's emerging roles in ER‐phagy and disease, these probes are timely tools. In 2022, Tolmachova et al., generated UFM1 activity‐based probes by converting recombinant UFM1 into a *C*‐terminal acyl hydrazide, which was then chemoselectively modified with electrophiles. A panel of probes was created, including α‐chloroacetyl, fumarate, epoxide, and alkyne derivatives [97]. α‐Chloroacetyl UFM1 showed remarkable selectivity and efficiency for covalently labeling the UFSP2. Besides UFSP2, the probe also labeled USP21, suggesting the cross‐reactivity of this DUB in UFM1 pathway.

### 4.6 URM1

URM1 (Ubiquitin‐related modifier 1) is often described as a “molecular fossil” Ubl that links the Ub system to prokaryotic sulfur transfer processes [98]. URM1 can act both as a protein modifier and as a sulfur carrier in tRNA thiolation. The URM1 conjugation cascade involves an E1‐like enzyme called UBA4 (which has a dual role in thiocarboxylating URM1). URM1 is *C*‐terminally activated by UBA4 forming a thiocarboxylate (CO‐SH) on URM1, and this thiocarboxylated URM1 can attach to lysines on target proteins without a conventional E2 or E3 [99, 100]. Notably, URM1's attachment to proteins may occur via a non‐enzymatic process triggered by oxidative conditions [101]. To date, no specific de‐URMylase enzyme has been identified. It is believed that URM1 modifications might be removed by general proteolytic turnover of the target or possibly a yet undiscovered isopeptidase.

Given this, ABPs for URM1 have not been a major focus, as there isn't a known URM1‐specific protease to profile. However, one could envision ABPs to study URM1's E1. A URM1‐Dha probe analogous to UFM1‐Dha could potentially trap UBA4's catalytic cysteine when it forms the URM1 thioester intermediate. Also, an ATP‐mimetic probe could be adapted for URM1. So far, one study used a “functional proteomics” approach with URM1 where a thiocarboxylated URM1 analog was used to capture interacting proteins [102, 103]. That is more of an affinity probe than a classical ABP, but it did link URM1 to certain pathways. In our recent study, we observed that URM1‐ACA, a fluorogenic probe, was cleaved in HEK293T cell lysates, indicating the presence of URM1‐processing proteases. Enzymatic activity was completely abolished by NEM pretreatment, suggesting the activity is mediated by cysteine‐dependent proteases, however, it's identity needs to be elucidated [104]. In conclusion, URM1 remains a special case in the Ub/Ubl family: lacking a dedicated protease, the typical ABP paradigm is less applicable, and research has focused on its conjugation mechanism instead.

### 4.7 Autophagy Ubls (Atg8/LC3/GABARAP, Atg12)

The autophagy pathway in eukaryotes employs two ubiquitin‐like systems: the Atg8 family (microtubule‐associated protein 1 light chain 3 (LC3A/B/C) and GABARAP subfamilies in mammals) which conjugate to the lipid phosphatidylethanolamine on autophagosome membranes, and Atg12, which conjugates to Atg5 (forming an E3‐like complex for Atg8 lipidation) [105]. These Ubls are essential for autophagosome formation. Their processing enzymes are unique: Atg4 cysteine proteases cleave pro‐Atg8 proteins to expose a *C*‐terminal Gly for lipidation and also can deconjugate Atg8 from PE. Atg12, on the other hand, is conjugated to Atg5 via a cascade (E1 Atg7, E2 Atg10) and forms a relatively stable conjugate; no dedicated Atg12‐deconjugating enzyme has been identified (the Atg12‐Atg5 conjugate is long‐lived).

ABPs for the Atg8 family were developed soon after the Ub probes. Hemelaar et al. included GATE‐16 (a GABARAP family member), GABARAP itself, and LC3 among their panel of VS probes [106]. These Ubl‐VS probes specifically labeled Atg4 proteases in cell lysates. For example, HA‐GABARAP‐VS reacted covalently with Atg4B (the major LC3/GABARAP protease in mammalian cells), allowing visualization of active Atg4B by gel shift. Such probes helped confirm that Atg4 activity can be inhibited by certain stimuli (e.g., nutrient conditions affecting redox state). More recent probes have used LC3B or GABARAP with propargyl warheads to enable fluorescence detection. Given Atg4's essential role and specificity (it only cleaves ATG8s and no other Ubls), these probes have a narrow target set, which is analytically convenient.

Atg12 is not known to be reversible (Atg12‐Atg5 is generally cleaved only by general proteases during protein turnover). Thus, there has been little need for an Atg12 isopeptidase probe. However, one could profile the E1 or E2 of Atg12 (Atg7 and Atg10) by using an Atg12‐ABP. To our knowledge, an Atg12‐Dha or Atg12‐VS probe has not been reported, possibly because Atg7/Atg10 activity can be inferred by other means. In any case, Atg7 has a catalytic cysteine for both the Atg8 and Atg12 systems, so a Ub E1 probe might label Atg7 as well. If specificity is desired, an Atg8‐ or Atg12‐derived ABP could be envisioned. In summary, the autophagy Ubl ABPs (Atg8‐family probes) fill a niche for studying Atg4 proteases. They confirmed Atg4's unique specificity and allowed assays for Atg4 inhibitors.

### 4.8 FUBI (MNSFβ)

FUBI is a ubiquitin‐like modifier co‐translated with ribosomal protein eS30 and released by dedicated cysteine proteases. [107, 108] Two human DUBs, USP36 (nucleolar) and USP16 (cytosolic), act as FUBI *C*‐terminal hydrolases, making them amenable to activity‐based profiling [109]. Full‐length FUBI‐vinyl sulfone (FUBI‐VS) and FUBI‐propargylamine (FUBI‐PA) probes, typically bearing *N*‐terminal detection/enrichment tags (e.g., HA, biotin, rhodamine), have been used to covalently trap active FUBI proteases in lysates and cell extracts (wild‐type enzymes label; catalytic Cys→Ala mutants do not). In side‐by‐side pulldowns, FUBI‐ABPs selectively enrich USP36 and USP16 with minimal cross‐labeling of other abundant DUBs that are robustly captured by ubiquitin probes (e.g., USP7, USP10), underscoring the narrow enzyme set that recognizes the FUBI surface [109]. Fluorogenic FUBI substrates (e.g., FUBI‐rhodamine110/Gly) corroborate this selectivity in kinetic assays, showing efficient turnover by USP36 and lower but measurable activity for USP16, while control DUBs are inactive. Beyond monomeric probes, an uncleavable di‐FUBI (triazole‐linked) ABP has been used as a chain mimic to discover FUBI binders in proteomes; notably, UCHL3 and IMPDH1 are captured, and di‐FUBI can inhibit UCHL3 activity toward Ub substrates, suggesting regulatory crosstalk at the binding level rather than productive FUBI hydrolysis [110]. Collectively, FUBI ABPs provide a selective handle on a restricted deFubiylase axis (USP36/USP16), enabling chemoproteomic mapping, validation of ribosome‐biogenesis steps involving FUBI‐eS30 processing, and orthogonal readouts to complement Ub/Ubl probe panels in complex samples.

The principles and warheads are the same as for ubiquitin. Combined with Ub and Ubl probes above, the arsenal of ABPs now covers nearly all ubiquitin‐like modifier systems with cysteine protease deconjugases.

## 5 ABPs FOR UBIQUITIN CONJUGATION SYSTEM (E1, E2, E3 ENZYMES)

While most early ABP efforts focused on DUBs and ULPs (i.e., the “deconjugation” side), recent years have seen significant innovation in developing probes for the Ub/Ubl conjugation enzymes: E1 activators, E2 conjugating enzymes, and E3 ligases. These enzymes often harbor catalytic cysteines that form thioester intermediates with Ub/Ubls, making them potential targets for covalent trapping. However, designing ABPs for them is more complex, as unlike proteases, they don't normally cleave a substrate bond that can be mimicked by a warhead [32]. Instead, ABP strategies have exploited key mechanistic steps in the conjugation cascade:

E1 enzymes: form an Ub‐adenylate intermediate and then a thioester with Ub (E1∼Ub). Probes can target either the E1's catalytic cysteine (to trap the thioester) or the Ub's *C*‐terminus during adenylation.
E2 enzymes: accept Ub from E1 to form an E2∼Ub thioester. Probes can be designed to capture E2‐bound Ub or to use E2 as part of a probe to trap E1 or E3.
E3 ligases: come in different types. HECT and RBR E3s form a catalytic thioester intermediate (E3Ub) and thus can in principle be labeled similarly to E2s. RING E3s do not form covalent intermediates; they facilitate direct Ub transfer from E2∼Ub to substrate, so ABPs for RINGs must employ alternate strategies (e.g., photo‐crosslinking when the E3 is engaged with E2∼Ub).

Below we discuss ABP developments for each class.

### 5.1 E1 Enzymes

Ub/Ubl‐activating enzymes (E1s) catalyze the first step of conjugation: they use ATP to adenylate the Ub/Ubl *C*‐terminus, forming an Ub‐AMP, then form a high‐energy thioester linking Ub to a catalytic cysteine on E1, releasing AMP. Humans have two Ub E1s (UBA1 and UBA6) and one or two E1s for each Ubl (e.g., UBA3/NAE1 for NEDD8, SAE1/SAE2 for SUMO, UBA7 for ISG15, UBA5 for UFM1, etc.) [111, 112]. Targeting E1s with ABPs is attractive for studying E1 inhibitor mechanisms (like the anticancer NEDD8 E1 inhibitor MLN4924). But E1s are challenging targets because their active‐site cysteine is only transiently acylated during catalysis, and the adenylate step has no covalent E1‐Ub linkage. Two main approaches have been achieved.

#### 5.1.1 Probes Targeting the E1 Catalytic Cysteine (Trapping Thioesters)

**FIGURE 10:**
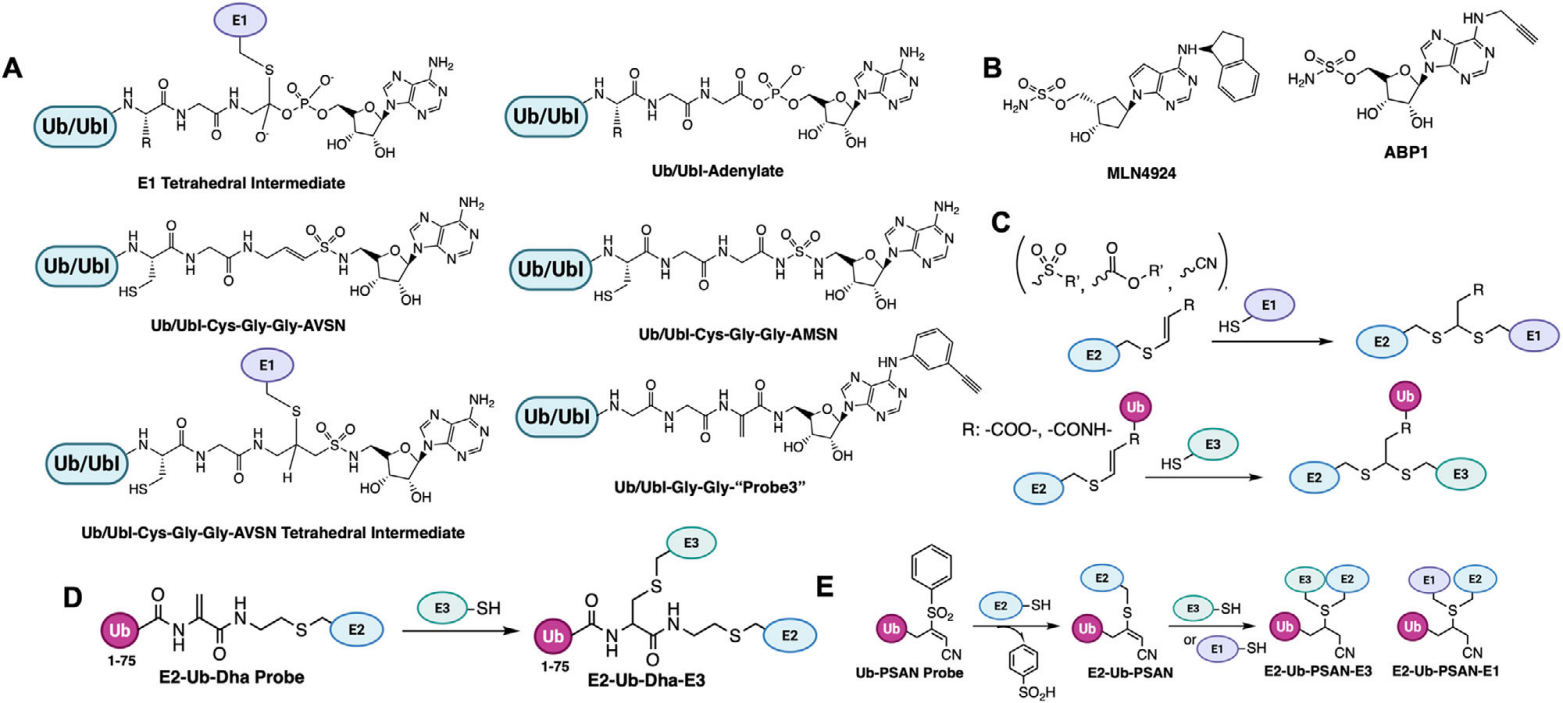
Activity based probes for ubiquitin conjugation system. (A) E1 probes that target E1‐catalytic cysteine (Ub/UbI‐E1 tetrahedral intermediate and Ub/UbI‐adenylate shown for comparison). Ub‐AVSN mimics Ub‐AMP and covalently traps the E1‐active site. E1 probes that mimic Ub/UbI adenylate complex such as Ub‐AMSN, an unreactive analog for Ub‐AMP. Ub‐Probe3 reacts similar to Ub‐AVSN, and it uses an alkyne handle for detection. (B) An E1 ABP1 probe which labels Ub and the NEDD8 inhibitor MLN4924 shares a related structural design and inhibitory mechanism. (C) Probes engineered for E1‐to‐E2 transthiolation step that covalently capture E1‐E2 intermediate and E2‐to‐E3 transthiolation step that covalently capture E2‐E3 intermediate. These probes are designed to intercept ubiquitin transfer from the E1 activating enzyme to the catalytic cysteine of E2 conjugating enzyme, also from E2 conjugating enzyme to the cysteine of HECT‐ or RBR‐type E3 ligases. By positioning a reactive electrophile at the ubiquitin *C*‐terminus or within the E2‐Ub conjugate, the probes form a covalent adduct with the active‐site cysteine during transthiolation. This strategy enables stabilization and capture of otherwise transient E2‐E3 intermediates, facilitating mechanistic and structural interrogation of ubiquitin ligation. (D) An E2‐Ub conjugate bearing a dehydroalanine (Dha) electrophile at the isopeptide junction mimics the native transthiolation intermediate and covalently captures the catalytic cysteine of HECT and RBR E3 ligases during Ub transfer, enabling activity‐dependent profiling and structural interrogation of E3 transthiolation states. (E) Ub‐PSAN constructs install a p‐sulfonylacrylonitrile (PSAN) electrophile to form covalent analogues of E1‐Ub‐E2 and E2‐Ub‐E3 transthiolation complexes, stabilizing otherwise transient catalytic intermediates for mechanistic and structural analysis of ubiquitin transfer.

An early example was a SUMO1‐AVSN probe: SUMO1 modified with an analog of adenylate (a chemical trap for the E1‐SUMO intermediate). This probe, SUMO1‐AVSN (Figure 10A), formed a covalent adduct with the SUMO E1 (SAE2/UBA2) by attacking a stable analog of the tetrahedral intermediate in the E1‐SUMO‐AMP complex [113]. In SUMO1‐AVSN, the acyl‐phosphate linkage of SUMO1‐AMP is replaced with a vinyl sulfonamide. Simultaneously, a SUMO∼AMP mimic incorporating an unreactive acyl‐sulfamide (SUMO1‐AMSN) was also developed (Figure 10A). The SUMO1‐AVSN complex adopt a “closed” state, reorganizing residues to catalyze transesterification. These results show that E1 enzymes remodel a single active‐site region rather than using separate catalytic centers for adenylation and thioester transfer. Importantly, such synthetic adenylate mimics enabled trapping of otherwise transient E1 states and have also been shown to inhibit E1 enzymes [114]. However, SUMO1‐AVSN had drawbacks: it required a non‐native sequence at SUMO's *C*‐terminus and had no convenient tag for detection. To improve on this, Statsyuk and co‐workers designed Ub(Dha)‐based probes for E1. By replacing the *C*‐terminal Gly of Ub with dehydroalanine, they created “Ub‐Probe3” (Figure 10A) which could form a covalent linkage to the E1 catalytic Cys upon thioester formation [115]. Crucially, this preserved the native sequence (no extra linker needed) and simplified synthesis. They incorporated an alkyne tag on the probe (attached to the adenine of ATP that gets bound) for detection. This method was generalized to other Ubls: they successfully made LC3‐Dha probes for Atg7, etc., demonstrating activity toward target E1s. One issue encountered was that an Ub‐Dha probe, once loaded onto E1, could sometimes be cleaved by DUBs in the lysate (e.g., USP5 releasing the Ub from E1). Thus, while E1 could be trapped, maintaining the adduct in a complex mixture required inhibitors or mutant background to avoid DUB action. Nonetheless, these direct E1 probes provided proof‐of‐concept for capturing E1∼Ub thioesters.

#### 5.1.2 Probes That Mimic the Ub‐AMP to Label the Ub (Substrate) Instead of the Enzyme

This ingenious strategy led to the small‐molecule ABP1 [116]. ABP1 is a bifunctional molecule that resembles the pharmacophore of MLN4924 (pevonedistat), a drug that forms a NEDD8‐inhibitor adduct in the NEDD8 E1 (Figure 10B) [117]. ABP1 contains an adenosine sulfamate motif that reacts with the E1∼Ub thioester to create a stable Ub‐AMP analog (a Ub linked via a sulfamate to the probe). In essence, ABP1 “captures” Ub after it has been transferred onto E1 by intercepting the thioester and generating a covalent Ub‐probe conjugate. This means the probe labels the Ub/Ubl (which remains bound to E1) and thus reports on E1 activity indirectly. ABP1 was designed to be pan‐specific: indeed, it can react with multiple E1 enzymes (Ub E1s, NEDD8 E1, SUMO E1, etc.) as long as the respective Ub/Ubl is present and activated. By including an alkyne handle, ABP1 allowed click‐attachment of fluorescent or affinity tags for readout. A major advantage of ABP1 is cell permeability. It is a cell‐permeable small molecule so that one can treat living cells and then detect Ub/Ubl‐ABP adducts inside cells. In practice, ABP1 has been used to measure target engagement of E1 inhibitors in cells: for example, cells treated with an E1‐active drug show reduced ABP1 labeling of that target, indicating occupancy. ABP1 itself (and analogs ABP2, ABP3) showed some E1 selectivity: one analog preferentially formed adducts with Ub and NEDD8 (over SUMO, etc.), implying it favored UBA1/UBA6 and NAE1 over other E1s [46]. ABP1's mechanism results in a N‐acyl‐sulfamate linkage between Ub and the probe, which interestingly can be hydrolyzed in acidic conditions. This is advantageous because after enriching the Ub‐probe adduct (e.g., via biotin‐click and streptavidin pulldown), one can release the Ub (and attached E1) by mild acid, simplifying proteomic analysis [118, 119].

**FIGURE 11:**
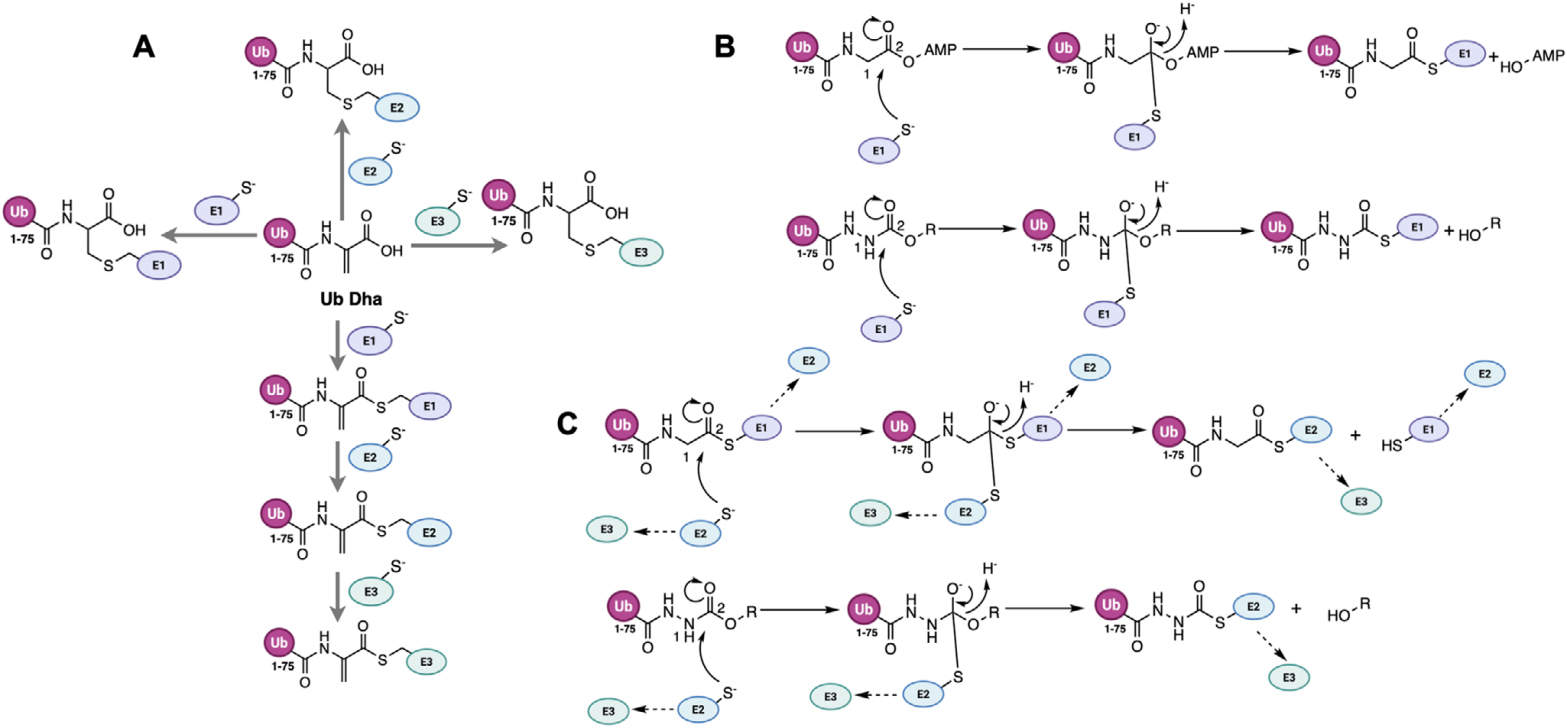
Ub‐Dha and ubiquitin azapeptide esters as activity‐based probes for E1‐E2‐E3. (A) Ub‐Dha pan cascade probe that undergoes Michael addition with catalytic cysteines across the pathway, covalently trapping E1, E2, and cysteine‐dependent E3 enzymes as stable Ub‐enzyme adducts. Upon adenylation by the E1 enzyme, Ub‐Dha is transferred to the active‐site cysteine of E1, forming a covalent adduct. Ub‐Dha can subsequently be relayed to E2 conjugating enzymes and further transferred to E3 ligases, thereby propagating through the ubiquitin activation and conjugation machinery. (B) Ubiquitin azapeptide ester probes that very closely mimics the E1 and (C) E2 and E3 ubiquitin tetrahedral transition state and targets the enzymes in a mechanism similar to native enzymatic catalysis which has been shown for comparison (Adapted with permission from Ref. [34], 2025, J. Am. Chem. Soc.).

Ub‐Dha (ubiquitin with a *C*‐terminal dehydroalanine) functions as an electrophilic mimic of native ubiquitin and can traverse the full E1‐E2‐E3 cascade (Figure 11A). Upon activation by E1, Ub‐Dha forms a thioester intermediate that can be transferred to E2 and E3 enzymes, ultimately reacting with their catalytic cysteines via Michael addition to yield stable covalent adducts. Ub azapeptide ester probes react with active‐site cysteines of E1, E2, and cysteine‐dependent E3 ligases via a transition state mimicking reactions catalyzed by these enzyme groups. The resulting thiocarbamate linkage very closely mimics the native E1‐AMP intermediate, Ub‐E1/E2/E3 thioester (Figure 11B,C). All reactions pass through a tetrahedral transition state that is potentially stabilized by an anion hole in the enzyme active site.

In summary, ABPs for E1 have evolved into powerful tools to study both the adenylation and thioesterification steps of Ub/Ubl activation. Direct E1‐targeting probes (like Ub‐Dha) trap the E1∼Ub intermediate but may need careful use to avoid DUB reversal. Substrate‐targeting probes (like ABP1) report on E1 activity in cells and have proven useful for inhibitor characterization. Together, they fill a critical gap in ubiquitin research, enabling the formerly invisible activation step to be monitored. It is worth noting that ABP1‐class probes do not distinguish which E1 enzyme is labeled (since multiple E1s use the same Ub), so one must parse the results (often by molecular weight of the Ub‐E1 band or by adding specific Ubls). In complex proteomes, distinct E1s can indeed be simultaneously labeled (e.g., UBA1 vs UBA6 vs UBA7, each transferring their cognate Ub or Ubl to ABP1). Despite these complexities, the versatility and cell‐based applicability of ABP1 represents a significant advance.

Finally, there is another clever ABP approach that indirectly profiles E1→E2 transfer activity: Stanley et al. developed probes consisting of an E2 enzyme with its catalytic cysteine mutated to an electrophile [120]. Specifically, an E2 (like UbcH7 or Ubc13) was engineered with a vinyl thioether handle on the active‐site Cys, so that when E1 attempts to transthiolate Ub onto this E2 (Figure 10C), the E1's cysteine attacks the electrophile on E2, forming a covalent E1‐E2 link. In cell lysates, an E2‐probe derived from UBE2N selectively labeled the Ub E1 (UBA1) it interacts with. This approach essentially captures the transient E1‐E2 thioester transfer state. It was used in ABPP assays to test E1 inhibitors competitively. Interestingly, these E2‐derived probes did not label any E3s in lysates, presumably because an E3 requires an E2∼Ub conjugate (not an empty E2) to bind productively [121].

### 5.2 E2 Enzymes

Although E2s themselves are less often the direct target of ABPs (since they act mainly as carriers of Ub), they have been used as components of probes to capture E1s (as above) or E3s (see below). A direct ABP for E2 activity could theoretically involve a small molecule mimicking an E3 or substrate that traps an E2∼Ub thioester. However, such generic E2‐trapping reagents are not well established. Instead, the focus has been on using engineered E2‐Ub conjugates as probes for E3s.

One might consider an E2's active site as a target: for example, a ubiquitin vinyl sulfone (Ub‐VS) can react not only with DUBs but also with the active‐site Cys of E2s under the right conditions [25]. If an E2 is loaded with Ub‐VS (via its E1), it could form a dead‐end E2‐Ub adduct. Indeed, some early studies reported that certain E2s became labeled by Ub‐VS in the presence of E1 and ATP, effectively trapping the E2∼Ub thioester as a stable conjugate (E2‐Ub linkage). However, these were not extensively exploited as deliberate probes.

The more strategic approach has been to synthesize E2∼Ub conjugates with built‐in traps for interacting partners. This is covered in Section 5.3 since it relates to E3 profiling. The first substrate‐like E2∼Ub intermediate mimics used to capture E3‐catalyzed transfer states were introduced by Pan et al. in their mechanistic/structural study of yeast Ubr1 [122]. Using yeast Ubc2 (Rad6) as the E2, they developed chemical strategies that mimic the initiation and elongation steps of ubiquitin transfer, enabling cryo‐EM visualization of Ubr1‐Ubc2‐Ub complexes with N‐degron substrates. These probes were used to study transient E3‐E2‐Ub interactions structurally. In effect, they serve as reagents to lock E2‐Ub in a particular state (e.g., “closed” conformation conducive to E3 binding) and then capture E3.

To summarize, E2 enzymes per se haven't been the direct focus of ABP development as isolated targets (since they usually act only when charged with Ub). Instead, E2‐based probes are typically designed in tandem with Ub to probe the next step in the cascade: ubiquitin transfer to E3 or substrate.

### 5.3 E3 Ligases (HECT, RBR, RING)

E3 ubiquitin ligases are the most diverse component of the conjugation cascade, numbering >600 in humans. They determine substrate specificity of ubiquitination. There are three major families: HECT and RBR ligases, which form a covalent E3Ub intermediate via a catalytic cysteine (similar mechanism to E2s), and RING/U‐box ligases, which function as scaffolds to bring E2Ub and substrate together but do not form a Ub linkage themselves (they have no active‐site cysteine). Designing ABPs for E3s requires accounting for these differences.

HECT and RBR E3s: These enzymes have active‐site cysteines that accept Ub from E2 in a transthiolation step, then transfer Ub to substrate [120, 121]. In principle, a HECT/RBR E3 can be trapped by a Ub‐based electrophile similar to how a DUB is trapped (since mechanistically it's the reverse of a deubiquitinase reaction: forming a thioester rather than breaking one). Indeed, conceptually one could use a *“suicide Ub”* that, once transferred to E3's cysteine, forms an irreversible bond. Early attempts likely included using Ub‐VS or Ub‐Br to trap HECTs, but success was limited as an E3 will not accept Ub from a probe unless it is presented by an E2 or a mimic thereof in a productive complex. The breakthrough came by extending the E2‐based probes discussed above: Pao et al., (2016) built on the E2‐probe concept by creating an E2∼Ub conjugate ABP with an internal electrophile [123]. Specifically, they generated a stable conjugate of UbcH7 (UBE2L3) with Ub, where the natural thioester bond was replaced by a non‐cleavable linkage, and a Michael acceptor was embedded in the Ub moiety. This probe, being a mimic of the E2Ub complex, could engage HECT/RBR ligases as a real E2Ub would. When a target E3 ligase attempts to accept Ub, it attacks the electrophile, resulting in a covalent E3‐probe linkage.

Using this approach, Pao et al. successfully labeled Parkin, a RBR E3 ligase implicated in Parkinson's disease, with a UBE2L3∼Ub ABP. The probe was equipped with a 6×His tag and an alkyne, allowing detection by fluorescence after click‐labeling. They showed that wild‐type Parkin in cell lysates was robustly labeled upon probe addition, whereas pathogenic Parkin mutants (from PD patients) showed much less labeling, correlating with their known loss of E3 activity. Furthermore, by treating cells with mitochondrial uncouplers to activate PINK1 (a kinase that activates Parkin), they demonstrated increased probe labeling of Parkin in wild‐type cells but not in cells harboring PINK1 or Parkin mutations. This exemplified how an ABP can serve as a reporter of E3 activation in a physiologically relevant context.

Since that report, similar E2 Ub ABPs have been applied to other HECTs and RBRs. They provide a modular platform: by changing the E2 component to one that pairs with a different E3, one can target different subsets of ligases. For instance, one could use an E2 specific for HECT NEDD4 family vs. one for RBRs, etc., each loaded with Ub and an electrophile. These probes have the advantage of specificity. They require the intact active conformation of E3 (with E2‐Ub engaged) to react, so that they are less likely to label off‐target cysteines. Indeed, Pao et al. found that without Ub attached, the E2 probe did not label E3s at all, which implies that E3s are only trapped when Ub is present to induce the proper binding orientation. Subsequent work has generalized this concept using structurally refined E2‐Ub probes that closely mimic native transthiolation intermediates and enable both proteomic and structural interrogation of HECT and RBR ligases. These probes have revealed transient transfer states, enabled crosslinking mass spectrometry, and provided cryo‐EM structures of E2‐Ub‐E3 complexes. To more faithfully capture transient ubiquitin‐transfer intermediates, several groups have developed near‐native E2‐Ub conjugates that mimic physiological transthiolation states and enable covalent capture of cysteine‐dependent E3 ligases. Xu et al. introduced an E2‐Ub‐Dha probe (Figure 10D) in which a dehydroalanine electrophile is positioned between ubiquitin and E2, enabling covalent trapping of the catalytic cysteine of HECT and RBR E3 ligases during transthiolation [124]. Horn‐Ghetko et al. developed an isopeptide‐bonded E2‐Ub mimic using a catalytically inactive UBE2L3 mutant (Cys→Lys), stabilizing a pre‐transition state E2∼Ub conformation that enabled structural interrogation of RBR ligase assemblies [125]. Liang et al. extended this concept by chemically synthesizing activity‐based E2‐Ub probes via sequential ligation strategies, introducing 2,3‐diaminopropionic acid (Dap) into E2 to create electrophile‐bearing constructs optimized for capturing E3 transthiolation intermediates [126]. Most recently, Kochańczyk et al. reported Ub‐PSAN probes (Figure 10E) that enable formation and stabilization of both E1‐Ub‐E2 and E2‐Ub‐E3 transthiolation analogues, providing unprecedented access to otherwise fleeting catalytic states for structural and mechanistic analysis [127]. Collectively, these probes represent a new generation of intermediate‐state ABPs that bridge mechanistic enzymology and structural biology to map ubiquitin transfer with near‐native fidelity.

RING E3s: Representing the largest class, RING‐type ligases (including multi‐subunit RING complexes like cullin‐RING ligases) do not form an Ub‐E3 intermediate. Instead, they facilitate direct transfer from E2Ub to substrate. This posed a significant hurdle for ABP design—there is no catalytic cysteine to target on a RING. However, RING E3s typically bind the E2Ub in a specific conformation (often termed “closed” conformation) that brings the donor Ub into proximity of the substrate. Researchers reasoned that if one could lock the E2Ub in that closed state and introduce a photoreactive crosslinker, one might capture the RING while it holds the E2Ub. This led to photo‐crosslinking activity‐based probes (photo‐ABPs) for RING E3s.

Sunil Mathur and colleagues engineered such probes: they used a Ub‐loaded E2 (such as UBE2D3.Ub) where (1) the thioester was isopeptide‐bonded (to prevent spontaneous hydrolysis) and (2) a photo‐crosslinker (like p‐benzoyl‐L‐phenylalanine, Bpa) was incorporated at a specific position on Ub [128]. The position was chosen such that in the closed E2Ub·E3 complex, the Bpa is adjacent to a surface on the RING domain. In the absence of an active E3, the E2Ub probe predominantly adopts an open conformation (Ub not near E2's binding interface). But when an active RING E3 binds, it stabilizes the closed conformation, bringing the Bpa into contact with the E3. UV irradiation then induces a covalent crosslink from Bpa to the RING protein. The crosslinked E3‐E2Ub complex can be detected by its shifted molecular weight or by specific antibodies.

Using such photo‐ABPs, Mathur et al. demonstrated UV‐dependent labeling of several RING E3s (RNF4, Cbl, etc.) only when they were in active conformations. In a follow‐up application, this technology was used to monitor the activity of RNF12 (RLIM), a RING E3 implicated in an X‐linked developmental disorder. The photo‐ABP robustly distinguished wild‐type RNF12 (which crosslinked upon UV, indicating active E3 function) from various patient‐derived RNF12 mutants that failed to crosslink, reflecting loss of activity. Moreover, these photo‐ABPs could be applied in cell extracts and even live‐cell contexts (e.g., in differentiated stem cells) to read out E3 activity changes. For RING and other E3 profiling, chemically tailored SUMO‐E2 conjugates incorporating photoreactive groups have been valuable. Bode and co‐workers synthesized SUMO1‐Ubc9 conjugates modified with a diazirine crosslinker and biotin tag, enabling UV‐activated capture of SUMO E3 ligases in ternary complexes [129].

In summary, RING E3 photo‐ABPs mark a creative leap in ABP design: they rely on conditional reactivity (light activation) and capture a protein‐protein interaction rather than a covalent intermediate. They underscore that ABPs need not always mimic a catalytic substrate; they can be engineered to trap transient complexes in an activity‐dependent manner. Photo‐ABPs, together with chemical ABPs for HECT/RBR, provide near‐complete coverage of the ubiquitination cascade enzymes.

## 6 Ub/Ubl Fluorogenic Probes

Fluorogenic substrates have long been indispensable tools for monitoring the catalytic activity of deubiquitinases (DUBs) and ubiquitin‐like protein specific (ULPs) proteases. These reagents consist of an Ub or Ubl protein conjugated at its *C*‐terminus to a small, fluorogenic leaving group, enabling direct, real‐time readouts of proteolytic activity.

The earliest and most widely used example is Ub‐AMC, in which 7‐amino‐4‐methylcoumarin (AMC) is conjugated to the *C*‐terminal Gly of ubiquitin. [130, 131]. Upon cleavage by an active DUB, AMC is released and fluoresces, providing a sensitive assay readout. Analogous Ubl‐AMCs (e.g., SUMO1/2/3‐AMC, NEDD8‐AMC, ISG15‐AMC, UFM1‐AMC) have been synthesized either commercially or via laboratory‐based efforts [96, 132, 133, 134]. The predominant synthetic route has relied on expressed protein ligation (EPL), where a recombinantly expressed Ub^1^^−^^7^ ^5^ or Ubl‐cGly‐intein fusion is converted into a thioester intermediate and subsequently ligated with Gly‐AMC [135]. While this platform has enabled countless studies of DUB/Ubl proteases, it faces important limitations. First, Gly‐AMC exhibits poor aqueous solubility, which complicates the ligation chemistry and results in reduced overall yields. Second, the Ub/Ubl‐intein fusions are often labile and difficult to purify, further restricting scalability. As a result, despite Ub‐AMC's prominence in biochemical assays, its use in high‐throughput screening (HTS) has been limited due to cost, low yields, and restricted substrate availability.

**FIGURE 12:**
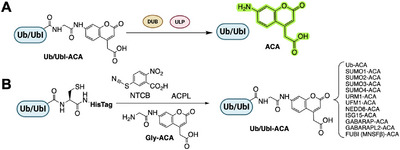
Newly developed Ub/Ubl‐ACA fluorogenic probes. (B) Schematic illustration of Ub/Ubl‐ACA probes as a next‐generation fluorogenic substrate for deubiquitinases (DUBs) and ubiquitin‐like protein specific proteases (ULPs). Enzymatic cleavage at the *C*‐terminus of the Ub/Ubl releases the ACA fluorophore, resulting in a measurable fluorescence signal. (B) Summary of engineered ubiquitin (Ub) and ubiquitin‐like proteins (Ubls) having Gly‐to‐Cys mutation at the terminal glycine position and a *C*‐terminal 6×His tag. Proteins include Ub, SUMO1‐4, URM1, UFM1, NEDD8, ISG15, GABARAP, GABARAPL2, and FUBI. These proteins bearing a *C*‐terminal cysteine undergo a nucleophilic substitution with Gly‐ACA in presence of NTCB, leading to the formation of desired Ub/Ubl‐ACA probes (Adapted with permission from Ref. [104], 2025, ACS Chemical Biology).

To overcome these challenges, we recently introduced a new class of fluorogenic probes in which AMC is replaced by 7‐amino‐4‐coumarinylacetic acid (ACA) [34]. This structural modification preserves the favorable spectroscopic properties of coumarin while greatly improving water solubility, stability, and synthetic accessibility. We previously developed the Activated Cysteine‐based Protein Ligation (ACPL) technique to enable site‐specific conjugation [136, 137]. It uses 2‐nitro‐5‐thiocyanatobenzoic acid (NTCB) to activate a cysteine residue, which then undergoes a one‐step exchange with an amine‐containing small molecule to yield the desired conjugate. Using this streamlined ACPL strategy, we generated Ub‐ACA (Figure 12) by reacting recombinant ubiquitin with Glycine‐7‐amino‐4‐coumarinyl acetic acid (Gly‐ACA) and extended the platform to multiple Ubls, including SUMO1‐4, NEDD8, ISG15, URM1, UFM1, GABARAPL1, GABARAPL2, and FUBI (MNSFβ) [104]. In contrast to Gly‐AMC, Gly‐ACA is readily water‐soluble, enabling efficient and scalable synthesis of ubiquitin‐ACA fluorogenic probes using the ACPL strategy. This improved solubility facilitates large‐scale laboratory preparation and substantially lowers the cost and practical barriers associated with the production of conventional Ub‐AMC substrates. The biochemical assays demonstrated that Ub‐ACA exhibits equal or superior enzymatic turnover compared to Ub‐AMC, while avoiding solubility bottlenecks. The substrate was readily cleaved by a broad panel of DUBs producing robust fluorescence signals with improved reproducibility relative to AMC substrates. Importantly, the enhanced solubility and simplified synthesis of ACA derivatives allowed preparation of diverse Ubl‐ACA reagents, enabling kinetic profiling of SENPs, NEDP1, USP18, UFSP2, ATG4, USP36 and other Ubl‐specific proteases.

A notable strength of the ACA platform is its ability to reveal biology that had remained opaque with legacy AMC reagents. First, SUMO4‐ACA, despite SUMO4's sequence idiosyncrasies—was cleaved efficiently by SENP1 at levels comparable to SUMO1‐3, indicating that the SENP recognition elements are conserved across all SUMO isoforms and that SUMO4 is more connected to canonical SUMO proteolysis than previously appreciated. Second, URM1‐ACA was robustly hydrolyzed in human cell lysates, and this signal was abolished by N‐ethylmaleimide, implicating one or more cysteine proteases as bona fide URM1‐specific proteases (deUrmylase)—arguably the first biochemical evidence for such activity. Together, these findings underscore how Ub/Ubl‐ACA substrates, by virtue of their streamlined synthesis and superior solubility, not only replace Ub/Ubl‐AMC in routine assays but also can facilitate High‐Throughput Screen (HTS), expand discovery space, uncovering unanticipated enzyme‐substrate relationships.

In addition to coumarin‐based substrates, rhodamine‐derived fluorogenic probes have emerged as valuable alternatives for Ub/Ubl enzyme assays. Compared to AMC, rhodamine fluorophores operate at longer excitation and emission wavelengths, reducing interference from compound autofluorescence and thereby minimizing false negatives in bioactive molecule screening. Recent examples include rhodamine‐based ubiquitin and ISG15 probes by Lb^pro^ ligation method reported by Wang et al. which demonstrate improved assay robustness and sensitivity for monitoring DUB and deISGylase activities [138]. Beyond deubiquitinases, fluorogenic ubiquitin probes have also been adapted to interrogate cysteine‐based E3 ligases. Notably, Berrocal et al. introduced a pro‐fluorescent ubiquitin probe, UbSRhodol, that undergoes signal unmasking upon transthiolation, enabling real‐time readout of HECT‐ and RBR‐type E3 activity [139]. Together, these advances highlight how red‐shifted fluorophores and pro‐fluorescent designs expand the scope of Ub‐based fluorogenic tools from DUB profiling to broader interrogation of ubiquitin cascade enzymes.

## 7 Comparative Analysis of Ub/Ubl ABPs

Across the spectrum of ABPs, ranging from those targeting DUBs to E3 ligases, their utility can be evaluated in terms of properties, performance, and application. A central consideration is the balance between specificity and breadth: while some ABPs function as broad‐spectrum tools labeling multiple related enzymes, others are designed with high selectivity for a single enzyme or a defined subset.

### 7.1 Breadth of Target Enzymes

Monoubiquitin‐based probes (Ub‐VS, Ub‐VME, Ub‐PA, Ub‐azapeptide esters) target dozens of DUBs across multiple families. They are excellent for profiling the global DUB activity of a sample in one experiment. In contrast, a diUb K63 probe, for example, will only label DUBs that accept K63 chains, giving a narrower readout. This is useful when one wants to focus on a particular signaling pathway. Similarly, ABP1 is broad in that it labels essentially all active E1 enzymes in a cell at once. If one needed to specifically profile only the SUMO E1, a different approach (such as a tagged SUMO that specifically traps SAE2) might be more appropriate. Thus, broad probes are great for discovery and global profiling, while selective probes are better for mechanistic studies of particular pathways.

### 7.2 Warhead Reactivity and Stability

VS and VME warheads offer a good balance of stability and reactivity and have become workhorse choices. The PA warhead is more reactive (especially in an enzyme active site environment) and uniquely cleavable by acid, which is a major plus for proteomic workflows. However, PA probes can in some cases label off‐target cysteines if used at high concentration; careful titration is needed. AOMKs, as noted, are very reactive but short‐lived in aqueous solution, making them less practical despite strong reactivity. Dehydroalanine warheads require more synthetic effort (nontrivial to incorporate), but they expand capability (e.g., enabling cascade probes and certain E1 traps). The mechanisms for enzyme trapping are different for various probes. Ub‐PA does a direct addition (cysteine attacks the alkyne carbon), Ub‐VS does a Michael addition, and azapeptide esters mimics the native transition state and targets the enzyme akin to native enzymatic catalysis. In practice, all yield irreversible adducts under assay conditions. The outcome might matter for downstream analysis. For example, Ub‐PA's thioether vs. Ub‐VS's thioether products are both stable but will have different mass spectrometry fragmentation patterns.

### 7.3 Sensitivity and Detection

Fluorescent ABPs (with TAMRA, Cy5, etc.) allow one‐step detection of active enzymes down to the low nanogram range on gels, often exceeding the sensitivity of immunoblots for a given DUB. This makes ABPs superb for detecting low‐abundance active enzymes. For example, Cy5‐Ub‐VME can reveal active USP7 in a cell extract even if no USP7 band is visible by Coomassie or immunoblot [56, 140]. On the other hand, one loses the ability to distinguish which enzyme is which if they run at similar positions; thus, follow‐up immunoprecipitation or mass spec is needed for definitive IDs. Affinity‐tagged probes require an extra blotting step but can directly identify the enzyme by Western blot if an antibody is available (e.g., probing an HA‐Ub‐VS‐treated lysate with anti‐USP14 to see if USP14 shifted). In complex proteomics, click‐chemistry tags (alkyne/azide) have proven invaluable, as they allow multiplexing: e.g., one can treat two samples with ABP1, click on a heavy vs. light isotope‐coded tag in each, and do quantitative MS (activity‐based proteomics with SILAC or isobaric labels) to compare active enzyme levels between conditions. Next‐generation Ub/UbI‐ACA fluorogenic probes breaks past the limitation of Ub‐AMC to provide more accurate, robust and real‐time kinetic readouts for the DUBs and ULPs that work in the relevant signaling pathway.

### 7.4 Selectivity and Off‐Targets

ABP specificity is generally governed by the recognition element. An Ub‐based probe will bind virtually any DUB that recognizes Ub. But as we saw, some DUBs can accommodate Ubls too (USP5 binding ISG15, UCHL1 binding NEDD8). This means a “Ub” probe might occasionally label a Ubl protease and vice versa. However, such cases are relatively rare and often reveal interesting biology (dual‐specificity enzymes). Off‐target labeling of proteins outside the intended enzyme class is also possible (e.g., a very reactive probe might alkylate abundant thiols in unrelated proteins). The literature reports few serious off‐target issues at working concentrations, because the probe's targeting element brings it to the enzyme's active site, hugely accelerating the reaction compared to random collision. The OTUB1 non‐catalytic labeling by Ub‐VS mentioned earlier is one of the few examples and is attributable to that particular enzyme's quirk (an accessible cysteine that reacted faster than the buried active‐site cysteine). Generally, one can control for off‐targets by including an excess of unlabeled competitor (e.g., pre‐incubate lysate with Ub‐aldehyde or a DUB inhibitor: true DUB bands will dim, non‐specific bands likely remain).

### 7.5 Physiological vs. Artifactual Readouts

ABPs measure the pool of enzyme that is active and accessible at the moment of lysis or labeling. An enzyme might be present but inactive due to phosphorylation or inhibitor binding—it will not be labeled, thus ABPs highlight the active subpopulation. For cell biology, this is a strength (monitoring regulation in real‐time) but also a caution: absence of a band doesn't always mean absence of the protein, only that it's not active under those conditions. A comparative example: an immunoblot might show a DUB is present in equal amount in two samples, but ABP labeling shows it is active only in sample A (perhaps due to upstream signaling). ABPs are therefore excellent for studying dynamic regulation. However, if an enzyme is tightly bound in a complex that sterically hinders the ABP, one might under‐report its presence. Some large protein complexes have inaccessible active sites that require conformational change to be labeled.

### 7.6 Integration With Structural Studies

Covalent ABP‐enzyme complexes have been crystallized to capture enzyme‐substrate analog structures. For instance, several DUBs have been crystallized with Ub‐VME or Ub‐PA covalently attached, revealing details of ubiquitin recognition [141, 142]. ABPs often act as irreversible inhibitors, stabilizing the enzyme in a “caught‐in‐the‐act” state amenable to crystallography. They can also be used in cryo‐EM for large complexes (e.g., an E3‐E2∼Ub complex crosslinked via photo‐ABP for structural visualization of the RING E3 engaged state).

### 7.7 Emerging Areas

The ABP concept is expanding beyond individual enzymes to entire pathways. The cascading Ub‐Dha probe is a prime example of a single reagent that can traverse the E1→E2→E3 pathway, labeling whichever enzyme is active along the line. This raises exciting possibilities: one could add a cascading probe to cells and then analyze by MS which E1, E2, or E3 got trapped—thus mapping the active ubiquitination network. Indeed, Mulder et al. suggested their Ub‐Dha probe (and analogs for SUMO, etc.) could be used for proteome‐wide profiling of active conjugation cascades. Another frontier is temporal control: photo‐ABPs for RING E3s show that adding a trigger (UV light) can give a snapshot of activity at a chosen time. Similarly, caged probes (inactive until uncaged by light or enzyme) have been explored to increase spatial/temporal precision.

Comparatively, Ub and Ubl ABPs have become as indispensable to ubiquitin research as antibodies are to traditional biochemistry. Each class (DUB vs. E1/E2/E3) required tailored strategies, but collectively they enable a 360° view of the ubiquitin signaling cascade in action. Table 1 provides a concise summary, which can guide researchers in selecting the appropriate probe for their enzyme or pathway of interest.

## 8 Conclusions and Outlook

Activity‐based probes have revolutionized the study of ubiquitin and Ubl enzymes by providing direct readouts of enzyme activity, not just abundance. Over the past two decades, continual innovation in probe chemistry has expanded these tools from the initial DUB‐directed reagents to a comprehensive suite that targets conjugating enzymes and even non‐catalytic ligase activities. In vitro and cellular studies using ABPs have mapped active DUB landscapes in various organisms, elucidated preferences for ubiquitin chain linkages, discovered regulatory post‐translational modifications on enzymes (via changes in probe reactivity), and helped validate mechanism‐based inhibitors for drug development. The concept of trapping an enzyme in its active state has proven general and powerful.

Looking ahead, several trends and challenges can be anticipated:

### 8.1 Increasing Selectivity and Versatility

While broad‐spectrum probes remain valuable, there is a growing need for selective ABPs to study individual enzymes in their native context (e.g., in live cells or specific organelles). This could involve designing probes that incorporate not just the primary recognition element but also secondary interactions (such as a substrate peptide or a particular chain type) to target one enzyme or a small subset. The “three generations” of DUB probes (S1‐only vs. S1+S1’ vs. S1+S2 targeting) illustrate this trajectory. In the future, a combination of protein engineering and chemistry might produce ABPs that discriminate between closely related isoforms or mutants—for instance, an ABP that labels *only* oncogenic USP7 and not wild‐type, by exploiting a mutation‐created pocket.

### 8.2 Live‐Cell ABPP and Imaging

A grand goal is to perform activity‐based profiling in live cells (or even in vivo). Some progress has been made, such as cell‐permeable ABP1 for E1s and cell‐penetrating fluorescent Ubl probes [112, 143] However, delivering large protein probes into cells without perturbing them is still an obstacle. Future strategies might include bio‐orthogonal precursors that are cell‐permeable and then ligate intracellularly to form the active probe or harnessing cellular expression systems (express a “proto‐probe” that can be activated by light or a small molecule to reveal the warhead). Genetically encoded photo‐crosslinkers (like unnatural amino acids introduced by amber suppression) could allow cells to biosynthesize something akin to a photo‐ABP inside them. These approaches could enable real‐time imaging of, say, DUB activation in response to a stimulus, providing spatiotemporal resolution that current endpoint ABPs lack.

**FIGURE 13:**
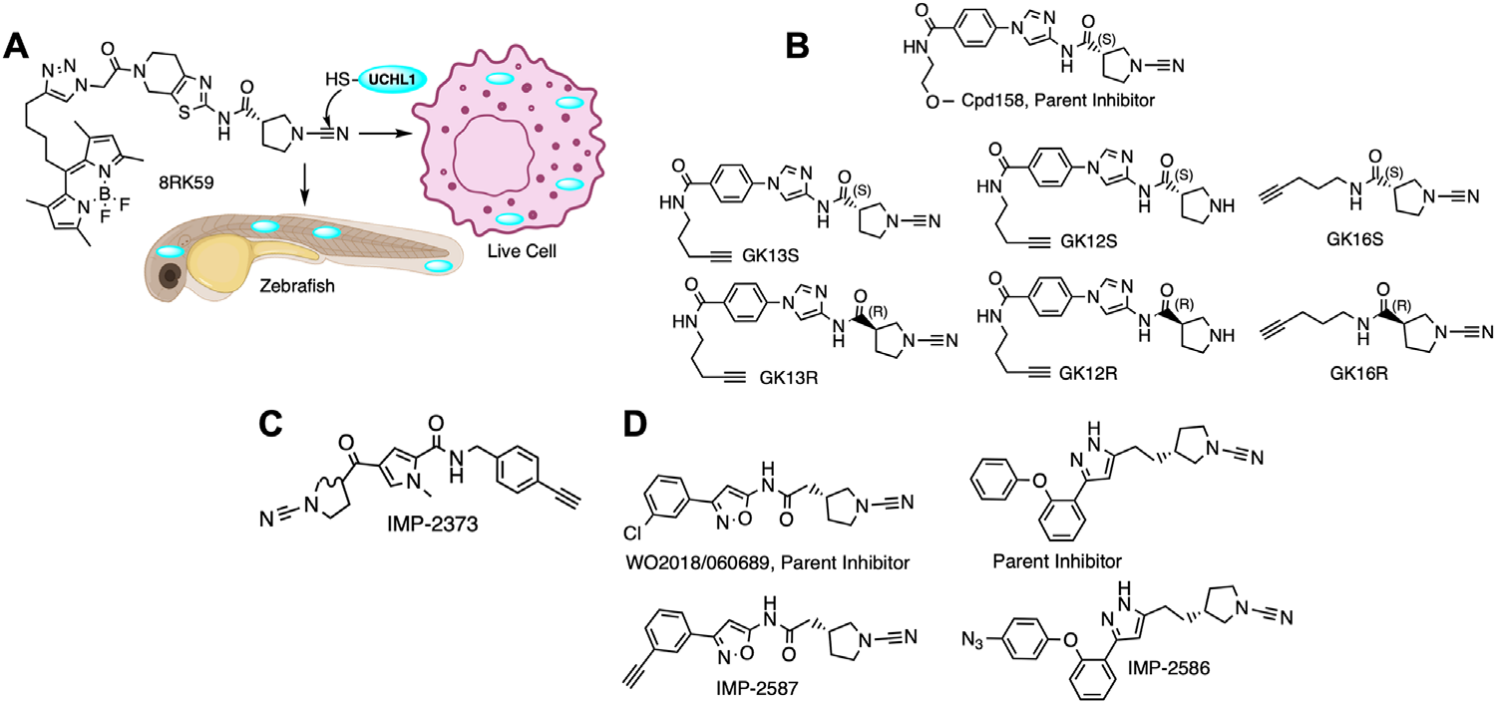
Small molecule‐based ubiquitin probes. (A) A cyanimide‐based small‐molecule inhibitor was derivatized with a fluorophore to generate a cell‐permeable ABP that selectively covalently labels the catalytic cysteine of UCHL1, enabling direct visualization of UCHL1 activity in live cells and zebrafish embryos (B) Nitrile‐based UCHL1 inhibitors are functionalized with orthogonal handles to create low‐toxicity ABPs that selectively and covalently label active UCHL1 in intact cells, enabling biorthogonal tagging and endogenous activity profiling (Adapted with permission from Ref. [145], 2022, Nature Communications). (C) Cyanopyrrolidine‐based small‐molecule ABPs covalently label catalytic cysteines of active DUBs and are optimized for chemoproteomic enrichment and competitive activity profiling to map DUB engagement and inhibitor selectivity. (D) A cyanopyrrolidine‐based ABP enables selective, inhibition of USP30 in living cells, providing a first‐in‐class small‐molecule tool to monitor endogenous USP30 activity.

In parallel with protein‐based probes, small‐molecule activity‐based probes (smABPs) have emerged as powerful tools for profiling deubiquitinase activity in living systems. Their compact size confers high membrane permeability, synthetic modularity, and facile derivatization, enabling the conversion of potent inhibitors into cell‐active probes. A seminal example is the cyanimide‐based UCHL1 probe developed by Kooij et al., (Figure 13A) which was derived from an inhibitor scaffold and subsequently functionalized with a fluorophore to enable direct visualization of UCHL1 activity in live cells and zebrafish embryos [144]. The probe covalently modifies the catalytic cysteine of UCHL1 and exhibits exceptional selectivity, as demonstrated by loss of signal upon UCHL1 knockdown. As shown in Figure 13B, Grethe et al. further expanded this strategy by developing nitrile‐based UCHL1 probes bearing orthogonal functional handles for biorthogonal tagging; notably, their compound GK13S selectively inhibits UCHL1 without impairing cell viability [145]. Conole et al. introduced cyanopyrrolidine‐based DUB probes (Figure 13C) optimized for chemoproteomic pull‐down and competitive activity profiling, highlighting the utility of this warhead class for selective covalent engagement [146]. More recently, Mondal et al. reported the first USP30‐selective small‐molecule ABP (Figure 13D), which operates in an activity‐dependent manner in intact cells and exploits a cyanopyrrolidine electrophile to achieve unprecedented selectivity [147]. Collectively, these studies demonstrate that smABPs provide a uniquely versatile and cell‐compatible platform for interrogating DUB activity with high specificity in physiologically relevant contexts.

### 8.3 Coverage of the Ubiquitin System

We now have ABPs for almost all cysteine‐based enzymes in ubiquitin/Ubl pathways. The notable gap is the metalloprotease DUBs (JAMM family). These require a different tactic since their mechanism (Zn^2+^‐dependent hydrolysis) won't be trapped by the electrophiles discussed. Designing an ABP for a JAMM might involve a coordinated inhibitor that irreversibly binds the Zn (like a hydroxamate with a photo‐crosslinker) [148, 149]. There has been some work on inhibitor‐based probes (e.g., hydroxamate inhibitors of AMSH linked to tags), but achieving specificity is tricky because many metalloproteases share inhibitor preferences [150]. This remains a challenge for the field: to profile active AMSH, BRCC36, etc., akin to how we profile active USP or OTU DUBs. A breakthrough here would complete the toolkit for DUBs.

### 8.4 Beyond Covalent: Proximity Labeling Approaches

Activity‐based probes have classically relied on irreversible covalent capture of catalytic cysteine residues. However, recent advances are rapidly expanding ABP design beyond classical covalent inhibition toward spatially resolved, hybrid, and real‐time ubiquitinomics technologies.

**FIGURE 14:**
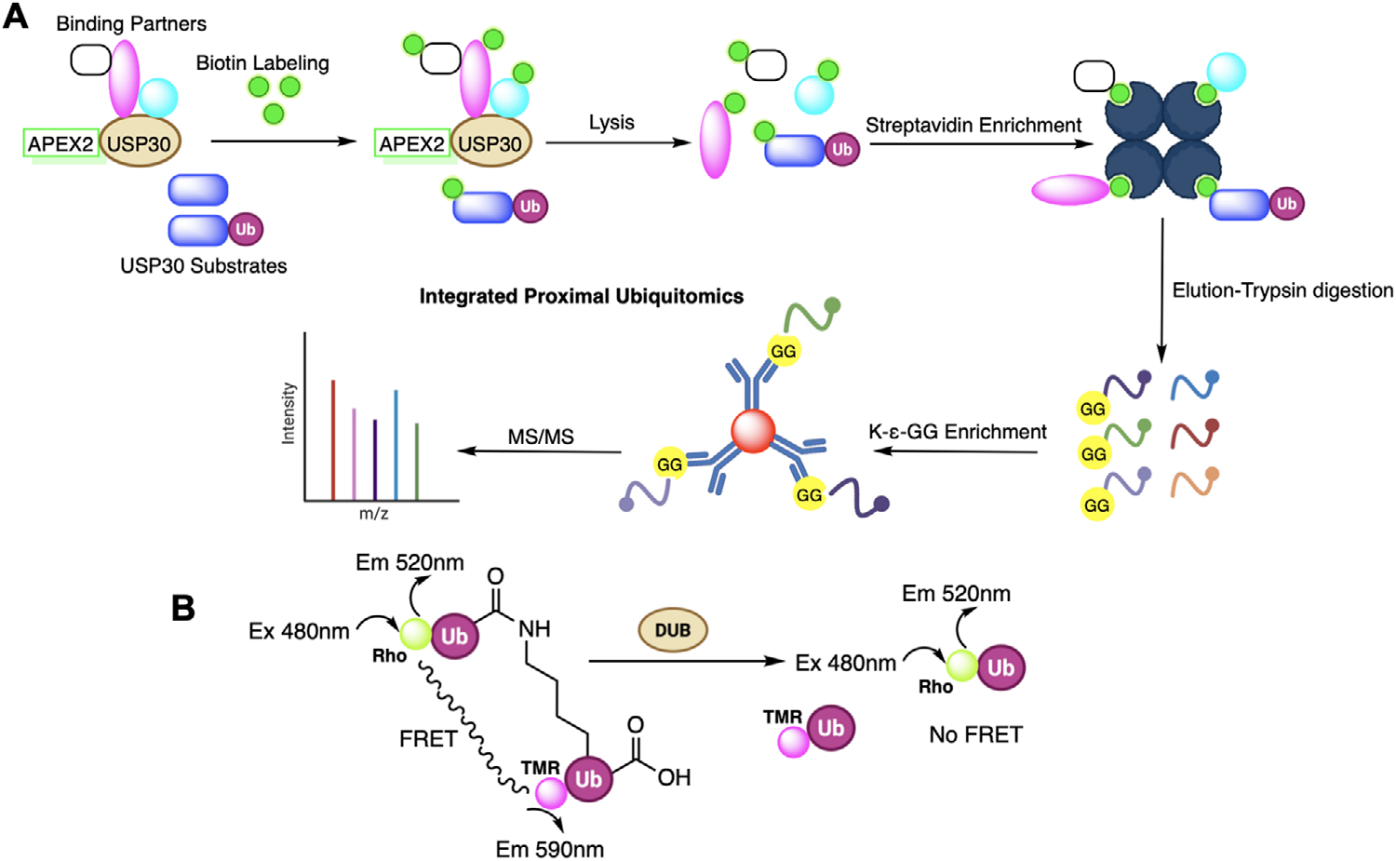
Newly developed and emerging labeling techniques. (A) Integrated Proximal Ubiquitomics method. Proximity labeling selectively biotinylates proteins within the immediate microenvironment of a DUB/E3 enzymes of interest. Cells are lysed and biotinylated proteins are captured by streptavidin affinity enrichment to isolate ubiquitination happening proximal to the labeled enzymes. Enriched proteins are digested with trypsin, and ubiquitinated peptides are further enriched by anti‐K‐ε‐GG antibody prior to LC‐MS/MS, enabling proteomic profiling of ubiquitination (Adapted with permission from Ref. [151], 2025, Cell Chemical Biology). (B) A FRET‐labeled diubiquitin substrate carries donor and acceptor fluorophores on the distal and proximal ubiquitin units, respectively, producing a strong FRET signal in the intact state. Cleavage by a deubiquitinase separates the fluorophores, leading to loss of FRET and enabling real‐time quantification of DUB activity and linkage specificity (Adapted with permission from Ref. [152], 2016, ChemBioChem).

A major recent breakthrough is the development of integrative proximal‐ubiquitomics profiling platforms. Damianou et al. (Cell Chem. Biol., 2025) introduced a strategy that couples APEX2 proximity biotinylation with di‐Gly (K‐ε‐GG) ubiquitin‐remnant proteomics, enabling identification of ubiquitination events (Figure 14A) occurring within the spatial neighborhood of a target enzyme in intact cells [151]. Applied to the mitochondrial DUB USP30, this platform provided a proximity‐restricted ubiquitination map that directly linked enzyme localization, catalytic activity, and substrate selection, establishing a new paradigm for spatially resolved ubiquitin signaling analysis. In parallel, photo‐crosslinking ABPs blur the boundary between classical covalent probes and proximity capture. These reagents exploit conditional, light‐triggered reactivity to trap transient E2‐Ub‐E3 assemblies and enable selective labeling of active RING ligases. Such designs demonstrate that ABPs can capture activity‐dependent protein‐protein interaction states rather than only catalytic nucleophiles. Looking forward, hybrid and reciprocal labeling chemistries are poised to further transform ubiquitin enzyme profiling. These include enzyme‐mediated tagging strategies in which catalytic turnover triggers release of a reactive fragment that subsequently labels the enzyme itself, as well as self‐immolative probes that generate a covalent tag only after substrate cleavage. These concepts integrate substrate recognition, proximity sensing, and irreversible trapping into a single molecular event, enabling fluorescent turn‐on and time‐resolved readouts of enzymatic engagement.

Finally, real‐time optical reporters are gaining increasing traction. FRET‐ and BRET‐based ubiquitination sensors provide dynamic, non‐invasive monitoring of ubiquitination and deubiquitination in living cells, complementing endpoint ABP profiling by reporting temporal changes in pathway activity. A diubiquitin substrate is engineered with a donor fluorophore on the distal ubiquitin and an acceptor fluorophore on the proximal ubiquitin, generating a high FRET signal in the intact state (Figure 14B) [152]. Upon cleavage of the isopeptide bond by a deubiquitinase, the two ubiquitin units dissociate, increasing the distance between fluorophores and resulting in a loss of FRET. The decrease in FRET provides a real‐time, quantitative readout of DUB activity and linkage specificity. Together, these emerging technologies position ABPs not merely as trapping reagents, but as central components of next‐generation ubiquitin signaling diagnostics.

### 8.5 Therapeutic and Diagnostic Applications

The fact that ABPs can identify active enzyme populations suggests possible use in diagnostics. For instance, ABP profiles of patient samples could indicate hyperactive vs hypoactive states of certain DUBs or ligases associated with disease. Already, Parkin activity in patient‐derived cells was assessed with an ABP. One can envision ABP‐based biomarkers for oncology (where a tumor might have elevated specific DUB activity that an ABP could detect in a biopsy lysate). Additionally, ABPs or their derivatives might be developed as therapeutic agents: an ABP is essentially an irreversible inhibitor with a specific targeting element. While large protein‐based probes are not drug‐like, small‐molecule versions (like ABP1 or improved variants) could potentially be leads for selective enzyme inhibition in vivo. For example, a rational design could convert a reversible inhibitor into an irreversible one by adding a warhead, creating a pharmacological ABP (somewhat analogous to how covalent drugs are designed for kinases or proteases).

In conclusion, activity‐based probes have opened a dynamic window into the otherwise elusive realm of ubiquitin signaling. They allow us to “spy” on the molecular machines that attach and remove ubiquitin as they operate. The synergy of synthetic chemistry, enzymology, and proteomics that ABP development embodies will continue to drive forward our understanding of the ubiquitin system. As we refine these tools to make them smarter, more selective, and more in vivo‐friendly, we edge closer to fully mapping and modulating the activity landscape of ubiquitin and Ubl networks in health and disease. The journey from the first HA‐Ub‐VS probe to Ub azapeptide ester probes, from sophisticated cascading and photo‐crosslinking probes to newly developed Ub/UbI‐ACA probes exemplifies the innovation at this chemistry‐biology interface. Future probes will no doubt bring even deeper insights, and perhaps clinical utility, demonstrating that the field of Ub/Ubl ABPs is very much active and brimming with possibilities.

## Conflicts of Interest

The authors declare no conflicts of interest.

## Data Availability

Data sharing is not applicable to this article as no new data were created or analyzed in this study.
